# Endothelium-specific sensing of mechanical signals drives epidermal aging through coordinating retinoid metabolism

**DOI:** 10.7150/thno.112299

**Published:** 2025-06-12

**Authors:** Xia Wu, Jiangming Zhong, Jingwei Jiang, Yi Zou, Dehuan Wang, Ziyan Chen, Mengyue Wang, Xinyu Shen, Zeming Li, Yang Xiao, Yuyan Yi, Fangqi Tang, Xiaoyu Long, Weiming Qiu, Qu Tang, Xiao Xiang, Xun Zhou, Mingxing Lei, Peng Shu, Qiang Zhou

**Affiliations:** 1 Department of Dermatology, Sir Run Run Shaw Hospital, Zhejiang University School of Medicine, Zhejiang 310016, China.; 2 HBN Research Institute and Biological Laboratory, Shenzhen Hujia Technology Co., Ltd., Shenzhen 518000, Guangdong, PR China.; 3 Key Laboratory of Biorheological Science and Technology of Ministry of Education & 111 Project Laboratory of Biomechanics and Tissue Repair, College of Bioengineering, Chongqing University, Chongqing 400044, China.; 4 Department of Burns and Plastic Surgery, Wuhan General Hospital of Chinese People's Liberation Army, Wuhan 430000, China.; 5 Three Gorges Hospital, Chongqing University, Chongqing 404000, China.; 6 Department of Dermatology and Cosmetology, Chongqing Hospital of Traditional Chinese Medicine, Chongqing 400021, China.

**Keywords:** aging, endothelial cells, retinol metabolism, mechano-chemical coordination

## Abstract

**Introduction:** Skin aging manifests as a systemic decay of intercellular mechano-chemical coordination. While vascular endothelial cells emerge as central orchestrators, their specific roles in sensing mechanical signals remain poorly understood.

**Methods:** To investigate age-related skin changes, we performed single-cell RNA sequencing (scRNA-seq) and spatial transcriptomics to analyze cellular proportions and differentially expressed genes (DEGs) across young, middle-aged, and elderly human skin samples. The mechanical properties of skin were quantified using photonic crystal cellular force microscopy (PCCFM) to compare Young's modulus between young and aged skin. Cell-cell communication networks, particularly interactions among fibroblasts, vascular endothelial cells, and epidermal cells, were deciphered via CellChat analysis in young versus aged groups. Functional validation of integrin receptors and the MK signaling pathway was conducted using aging mouse models and skin organoid systems. Age-associated biomarkers were identified through immunofluorescence staining, hematoxylin-eosin (HE) staining, and RT-qPCR. RNA-seq further screened downstream targets of the MK pathway. Skin organoid cultures were employed to validate the rejuvenating effects of retinol metabolites.

**Results:** Here we revealed that mechanoresponsive endothelial cells drive skin aging by orchestrating a tripartite axis (fibroblast-endothelial-epidermal) via integrin-mediated mechano-transduction that modulates retinoid metabolism. First, we found that reduced extracellular matrix (ECM) expression by fibroblasts weakens integrin-mediated interactions with endothelial cells, leading to a decreased number of endothelial cells and thinner skin during aging. Then, attenuated endothelial cells-derived MDK signaling to SDC4 in basal cells results in declined basal cell retinol metabolism, a process essential for maintaining skin homeostasis and regeneration. Using our established skin organoid model, we demonstrated that adding retinol metabolites can rejuvenate skin cells with better structural and functional integrity.

**Conclusions:** These findings highlight the intricate intercellular dynamics that underlie skin aging and shed light on the previously underexplored role of mechano-sensitive endothelial cells in this process. Aging as an endothelial-specific coordination failure with other cells in the skin and potentiates developing combinatorial mechano-metabolic intervention strategies to restore tissue-level rejuvenation.

## Introduction

Vascular aging constitutes a prior hierarchical orchestrator of systemic senescence, with emerging evidence suggesting its temporal precedence over peripheral tissue degeneration. Preserving vascular homeostasis may thus confer organism-wide geroprotection 1. Intriguingly, skin aging propagates senescent phenotypes across organ systems 2, positioning dermal microvascular aging as a critical nexus for understanding systemic aging mechanisms. While mechanotransduction dynamics govern endothelial homeostasis, both hypo- and hyper-mechanical stimulation disrupt vascular equilibrium, highlighting mechanomodulation as a strategic anti-aging target. Current paradigms predominantly focus on macrovascular mechanobiology in atherogenesis 3, 4, leaving cutaneous microvascular mechanosensing to be substantially underexplored.

Tissue-specific mechano-responses emerge through integrin-mediated ECM interactions during angiogenesis. Collagen-integrin binding initiates signaling cascades regulating endothelial migration and matrix remodeling 5. The dermal ECM's biophysical properties notably stiffness and topography mediate vascular structural support and fibroblast dynamics 6. Age-related ECM degradation disrupts endothelial mechanostasis by altering Sdc4/Vegfr/Piezo1-mediated sensing 7, while matrix stiffening impedes vascular morphogenesis by inducing dysfunctional endothelial spreading 8. These mechanical insults converge with p53/p21-mediated cell cycle arrest to drive endothelial senescence and pro-inflammatory secretome development 9.

Understanding the localization and environment of senescent cells, as well as their interactions with neighboring cells is crucial for advancing our understanding of the drivers of senescence and may help identify potential intervention targets 10. In aged skin, increased matrix stiffness in the dermal layers contributes to disrupting the dynamic microenvironment of vascular endothelial cells 8, 11-13. During angiogenesis, endothelial cells secrete matrix metalloproteinases (MMPs) to degrade the ECM, creating space for neointimal extension 14, 15. However, increased ECM stiffness alters the morphology and behavior of endothelial cells, causing them to shift from a spindle shape to isotropic spreading. This change hinders their ability to assemble into multicellular structures or rearrange into vascular cords 8, 16, 17.

In addition to the mechano-chemical interactions with dermal cells, vascular endothelial cells also influence epidermal development and renewal through paracrine signaling 18. Under pathological conditions such as diabetes, the secretory behavior of vascular endothelial cells is disrupted, leading to failure in epidermal regeneration 19. Recently, retinol has emerged as a promising agent for promoting skin regeneration and mitigating aging. Specifically, retinol enhances epidermal thickness by activating epidermal stem cells and promoting the proliferation of epidermal keratinocytes. This process involves the activation of the c-Jun transcription factor, which stimulates keratinocyte proliferation and significantly increases epidermal thickness 20. Furthermore, retinol-induced skin vascularization enhances blood circulation within the skin, creating a more favorable microenvironment for keratinocyte proliferation and facilitating the activation of skin fibroblasts. The proliferation of epidermal keratinocytes can, in turn, promote dermal vascularization by increasing the expression of vascular endothelial growth factor. These processes—keratinocyte proliferation, endothelial cell growth, and dermal fibroblast activation—are interrelated, forming a mutually reinforcing environment. This interplay may underlie the renewing role of retinol metabolism in the aging process of human skin 21. However, the specific tissue-level sensing of mechanical cues between these cells that drive skin aging remains poorly understood.

In this study, we employed an integrative approach, combining single-cell RNA sequencing (scRNA-seq), spatial transcriptomics, aging murine models, and skin organoid systems, to delineate the mechano-chemical signaling networks among fibroblasts, endothelial cells, and basal epidermal cells in aged skin. Our comprehensive analysis reveals that attenuated mechano-transduction due to decreased ECM-Integrin interaction between fibroblasts and endothelial cells led to declined structural and functional skin integrity during aging. In addition, we newly identified that crosstalk between endothelial cells and basal epidermal cells via the MDK-SDC4-RBP1 axis orchestrates retinol metabolism, which is essential for maintaining epidermal homeostasis. These findings not only elucidate the dual regulatory role of endothelial cells in transducing both mechanical and biochemical signals within aging microenvironments but also pave the way for novel anti-aging strategies. Specifically, targeting retinoid metabolism and mechano-transduction pathways may restore the skin regenerative capacity, offering promising avenues for future therapeutic interventions.

## Results

### Fibroblast-mediated ECM stiffening impairs mechano-adaptive responses in the aging endothelium

**Figure 1:**
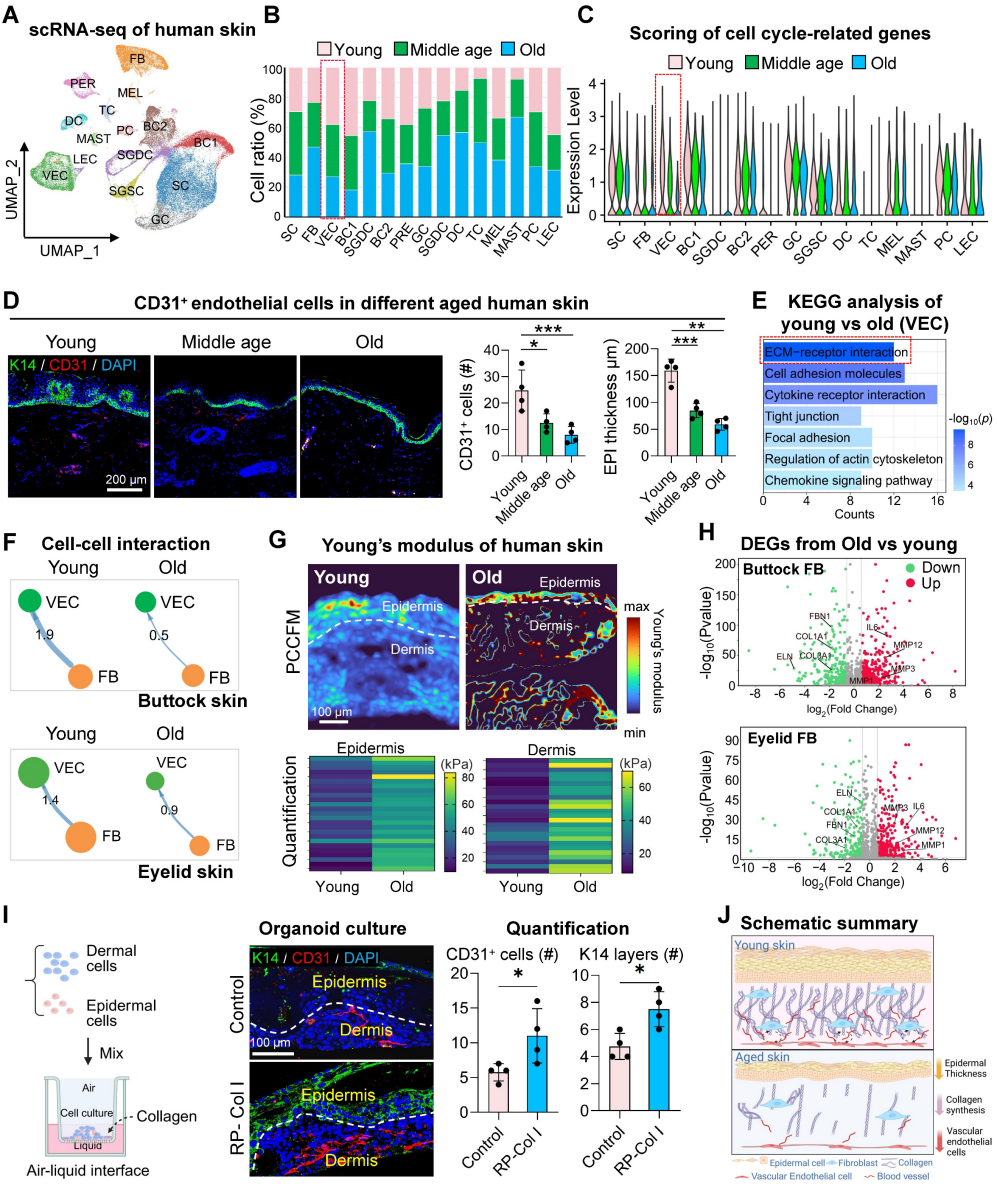
** Fibroblast-driven ECM stiffening disrupts endothelial mechanoadaptive capacity during aging.** A. UMAP visualization of integrated scRNA-seq data from human young, middle-aged, and aged buttock skin. B. Comparative analysis of cellular subset proportions in young, middle-aged, and old buttock skin. Red boxes indicate the age-dependent changes in the proportion of vascular endothelial cell (VEC) clusters, highlighting a decline in VEC abundance with advancing age. C. AUCell-based gene set enrichment scores for proliferation-associated genes across cellular clusters of human buttock skin. Red boxes highlight the age-related changes in proliferation gene scores within VEC clusters, demonstrating a significant reduction in proliferative capacity in aged VECs. D. Left: Representative immunostaining images of K14 (basal keratinocytes marker) and CD31 (endothelial marker) in human chest skin (n = 4). Right: Quantitative analysis of CD31⁺ cell density and epidermal thickness. Data are presented as mean ± SEM; *p < 0.05, **p < 0.01, ***p < 0.001, indicating significant age-related decreases in both parameters. E. KEGG pathway enrichment analysis of differentially expressed genes (|log₂FC| > 1, adjusted p < 0.05) in VECs between aged and young buttock skin. Key pathways related to ECM organization and angiogenesis are highlighted, showing significant downregulation in aged skin. F. Comparative analysis of CellChat-predicted interaction strength between FB and VEC in young versus aged skin. Top panel depicts buttock skin communications; Bottom panel showcases eyelid skin interactions. G. Young's modulus heatmaps of aged and young skin. Upper: Representative PCCFM images showing Young's modulus heatmaps of aged and young skin (n = 3 donors). Lower: Quantitative analysis of Young's modulus in epidermal and dermal regions (n = 25 pixels per group), demonstrating increased stiffness in aged skin. H. Volcano plots depicting differentially expressed genes (|log_2_FC| > 1, adjusted p < 0.05) in dermal fibroblasts comparing young versus aged skin. Top panel: buttock skin analyses; Bottom panel: eyelid skin profiling. I. Immunofluorescence of K14 and CD31 in Collagen-treated skin organoids. Left: Architectural schematic of skin organoid culture process. Middle: Representative immunofluorescence images of K14 and CD31 expression in skin organoids treated with recombinant type I murine collagen, n = 4. Right: Quantification of CD31⁺ cell density and epidermal thickness. Statistical significance: *p < 0.05 indicating enhanced vascular and epidermal integrity upon collagen treatment. J. Schematic representation of age-related changes. This diagram illustrates age-related declines in VEC numbers, collagen content, and epidermal thickness.

The skin which contains multiple tissues serves as a paradigm model for elucidating the complex interplay among diverse cell types within the aging microenvironment. To dissect intrinsic aging from photoaging, we analyzed two skin models: UV-protected buttock and photoexposed eyelid tissues. This dual-system strategy separated chronological aging dynamics (cellular crosstalk and transcriptional remodeling) from photoaging-induced ecosystem disruptions. We validated these findings using scRNA-seq datasets of human buttock (GSE274955) and eyelid (HRA000395) skin 22. For buttock skin, Seurat-based clustering of 31,549 cells (young: 23y; middle-aged: 53y; old: 85y) identified 15 subclusters via marker genes: spinosum cells (SC), fibroblasts (FB), vascular endothelial cells (VEC), basal cells (BC1/2), pericytes (PER), granulosum cells (GC), secretory/dermal sweat gland cells (SGSC/SGDC), dendritic cells (DC), T cells (TC), melanocytes (MEL), mast cells (MAST), proliferative cells (PC), and lymphatic endothelial cells (LEC) (Figures 1A, S1A-B). Parallel analysis of eyelid skin (young:18-23y; middle-aged:44-48y; old:70-76y) resolved 60,378 cells into 14 conserved subclusters (Figure S1C), enabling direct cross-tissue comparisons of age-related cellular reorganization. Subsequent comparative analysis of the relative proportions of each cell cluster revealed a marked decline in the prevalence of VEC and immune cells with aging in buttock skin (Figure 1B), underscoring the susceptibility of these cell types to the aging process. The expression of cell-cycle-related genes, which serves as a proxy for cellular youthfulness 23, was assessed via AUC scoring. This analysis revealed a significant age-dependent reduction in the average expression levels of these genes, particularly within the VEC cluster (Figure 1C), indicative of stagnation in endothelial cell proliferation concomitant with skin aging. Quantitative assessment of eyelid skin revealed age-dependent declines in vascular endothelial cell (VEC) proportions and concomitant reductions in cell cycle-associated gene scores (Figures S1D-E). Strikingly, parallel scRNA-seq profiling of groin skin demonstrated conserved depletion of VEC populations in aged cohorts, accompanied by diminished proliferative transcriptional activity (Figures S1F-H). These pan-tissue findings establish vascular endothelial attrition and cell cycle dysregulation as mechanistically conserved hallmarks of cutaneous aging across anatomically distinct skin regions.

Immunostaining, H&E staining, and statistical analysis of human skin across different age groups further corroborated these findings, demonstrating a pronounced decrease in CD31-positive cells within the dermis and a notable thinning of the epidermal layer during aging (Figure 1D, Figure S1I). To gain deeper insights into the transcriptional alterations associated with aging in VECs, we employed the *subset* function of the Seurat R package to isolate VEC clusters from both young and old groups. Subsequent KEGG enrichment analysis of genes downregulated in aged VEC clusters revealed a pronounced suppression of pathways related to "ECM organization" and "ECM structure organization" in the elderly group (Figure 1E). This suggests that the aging process in VECs may be closely tied to their diminished interactions with the ECM.

**Figure 2:**
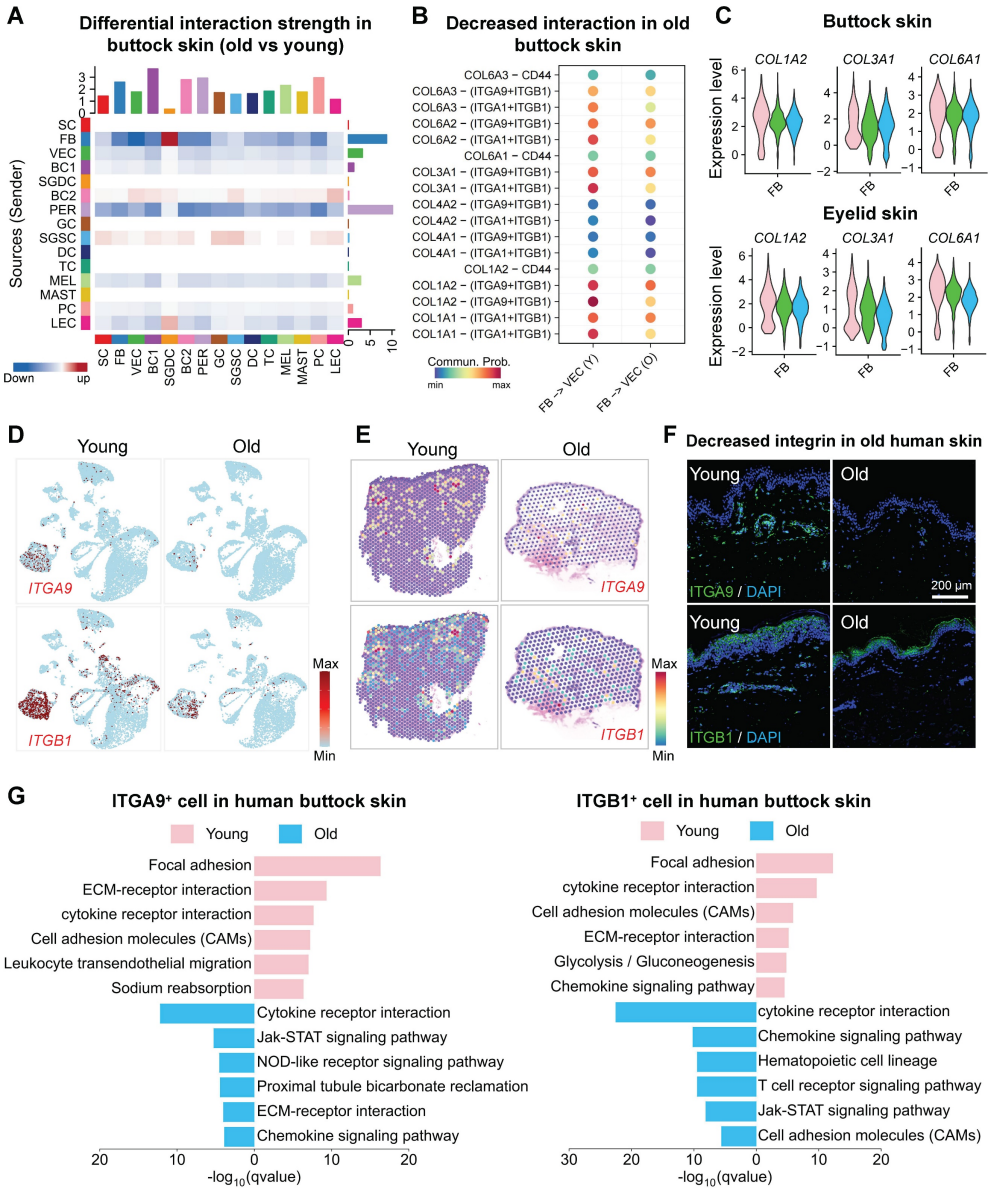
** Fibroblast-endothelial crosstalk attenuation compromises cutaneous vascular regeneration during aging.** A. Heatmap showing changes in cell-cell interaction strength between aged and young human skin. Red indicates upregulation in the aged group, blue indicates downregulation in the aged group. B. Dot plot illustrating differential Collagen-mediated interaction intensities between FB and VEC in young versus aged cohorts. C. Violin plots depicting age-dependent variations in collagen gene expression across young, middle-aged, and aged cohorts. Upper panel: Buttock skin, Lower panel: Eyelid skin. D. Feature plots showing the expression of ITGA9 and ITGB1 in young and aged buttock skin. E. ImageFeaturePlot visualization of spatial transcriptomic data depicting ITGA9 and ITGB1 expression in young and aged human skin. F. Representative immunostaining images of ITGB1 and ITGA9 expression in young human skin (n = 3 biological replicates). G. EGG pathway enrichment analysis of highly expressed genes in ITGA9⁺ and ITGB1⁺ VECs from young (upper panel) and aged (lower panel) buttock skin.

To explore this hypothesis, we leveraged the ECM-Receptor mode of CellChat 24 to analyze changes in cell-cell communication between young and aged skin. Our computational ligand-receptor analysis revealed a conserved age-associated decline in FB-VEC crosstalk, quantified through both sun-protected (buttock) and photoexposed (eyelid) skin models (Figure 2D-E). This pan-tissue attenuation of stromal-vascular signaling networks suggests microenvironmental uncoupling as a fundamental mechanism underlying cutaneous aging. Mechanosensitive receptors on the cell membrane, such as integrins and cadherins, play a pivotal role in transducing mechanical signals into biochemical signals through mechano-adhesion, thereby activating a cascade of intracellular responses 25. To investigate this process, we curated a gene set associated with biomechanical signal transduction based on a literature review (Figure S1J). Using AUCell, we evaluated the expression of these mechanoreceptor-related gene sets across age groups in human skin. Our analysis revealed that the expression of these gene sets was relatively conserved in VECs but exhibited a marked decline with advancing age (Figure S1K).

Given that the composition of the ECM can influence tissue stiffness, we employed photonic crystal cellular force microscopy 26 to assess changes in matrix stiffness between aged and young human skin. We observed a significant increase in the elastic modulus of aged skin (Figure 1G, Figure S1L), which may be closely linked to alterations in ECM composition. These findings suggest that fibroblasts may modulate endothelial cell aging by remodeling the ECM.

To elucidate how fibroblasts influence ECM changes in the aging skin microenvironment, we compared gene expression profiles of FBs between old and young groups from buttock skin or eyelid skin. The expression of senescence-associated secretory phenotype (SASP)-related genes was significantly elevated in FBs from the elderly group, whereas genes associated with ECM organization were more highly expressed in the young group (Figure 1H). To further explore the impact of ECM composition on cellular behavior, we utilized our established skin organoid system 27, 28. By supplementing the system with recombinant type I collagen, we investigated its effect on VECs. Surprisingly, treatment with 100 µg/mL collagen resulted in a significant increase in the number of CD31-positive cells within the organoid system (Figure 1I). Collectively, these results indicate that ECM-receptor interactions between FB and ECs are compromised in aged skin, potentially contributing to the stagnation of endothelial cell proliferation (Figure 1J).

### Age-related decline in fibroblast-endothelial cell interactions impairs vascular renewal in the skin

Fibroblasts may influence vascular renewal through interactions between the ECM and mechanosensitive receptors on endothelial cells 29, as well as through the secretion of pro-angiogenic factors. To delineate the dynamic remodeling of ECM-receptor signaling networks during aging, we performed CellChat communication analysis, revealing hierarchical degradation of mechanosensing capabilities in the skin microenvironment. The FB-VEC communication network exhibited systemic attrition with aging, where collagen-mediated mechanotransduction signals showed progressive deactivation in sun-protected regions (buttock skin, Figure 2A, S2A-B) and synchronized deterioration in photoexposed areas (eyelid skin, Figure S2C-D). Molecular pathway deconvolution identified specific impairment in the coordinated signaling capacity between COL1A2 and the integrin receptor complex (ITGA9/ITGB1), with spatial topological reorganization of this axis marking a critical rupture point in ECM-receptor crosstalk (Figure 2B, S2E). To investigate age-related changes in collagen expression, we employed violin plots to compare the expression of collagen-related genes COL1A2, COL3A1, and COL6A1 within the fibroblast cluster. The results revealed a significant age-dependent decline in the expression of these key collagen genes (Figure 2C). Comparative analysis of ITGA9 and ITGB1 expression across age groups by scRNA-seq and spatial transcriptomic analysis (Figure 2D-E, Figure S2F-G) and immunostaining (Figure 2F) demonstrated a significant age-related decline in the VEC cluster. Enrichment analysis of ITGA9 and ITGB1-positive endothelial cells in young and aged skin revealed that young VECs were primarily associated with ECM-receptor interactions, whereas aged V ECs showed enrichment in pathways related to cell adhesion and apoptosis (Figure 2G, Figure S2H). These suggest that the reduced expression of integrin receptors on aged VECs may impair their interactions with fibroblasts, leading to diminished self-renewal capacity.

### Integrin receptor activation promotes vascular remodeling and tissue rejuvenation in aged skin

**Figure 3:**
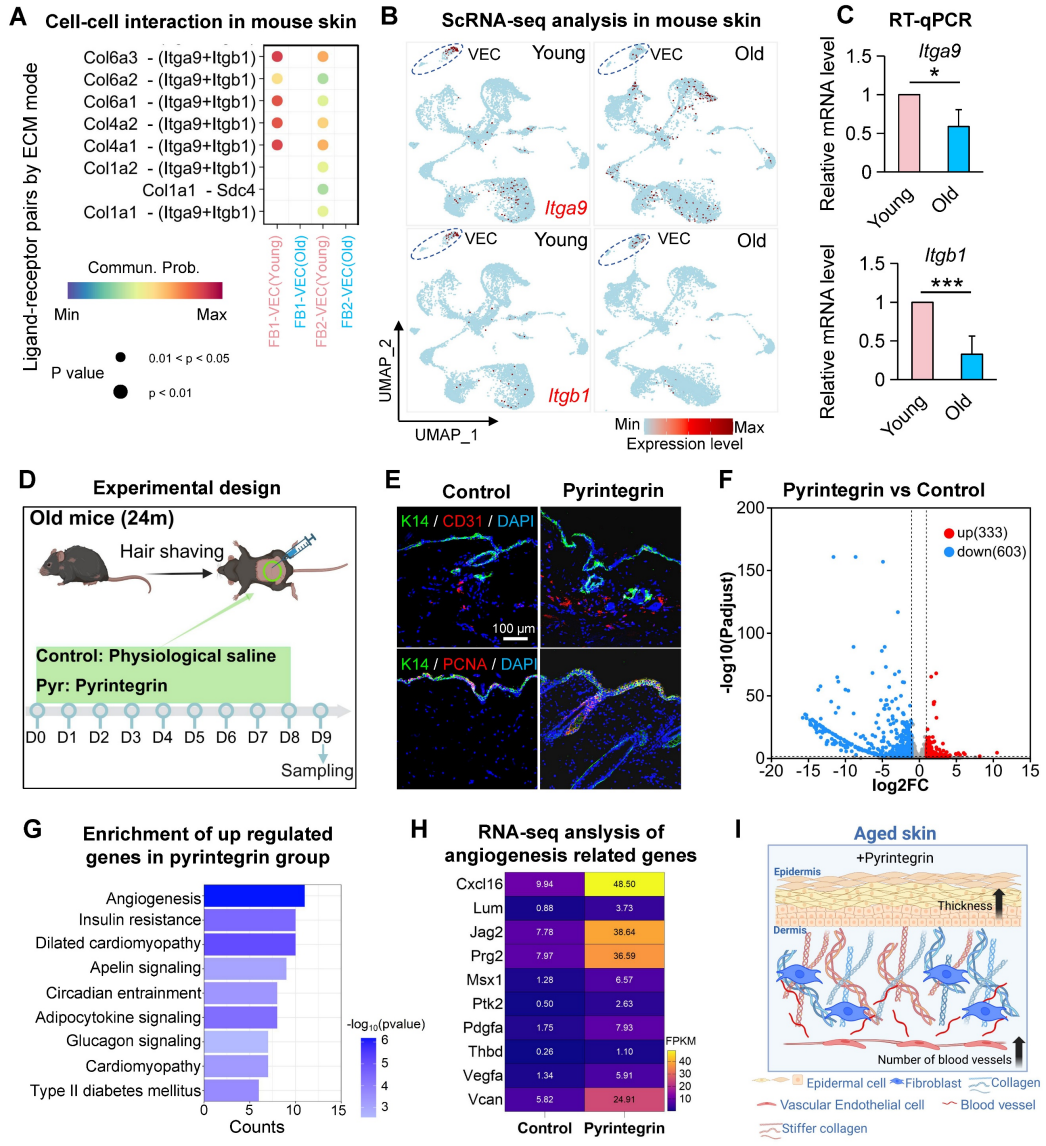
** Integrin-mediated mechanoactivation drives vascular remodeling and dermal rejuvenation in aged skin.** A. Bubble plot showing the changes in interactions between FB1 and FB2 with VEC in the scRNA-seq data of young and old mouse skin. B. FeaturePlot showing the expression of Itga9 and Itgb1 in the scRNA-seq data of dorsal skin from young and old mice. C. Bar graph showing the changes in relative expression levels of Itga9 and Itgb1 in the dorsal skin of young and old mice. N = 3, *p < 0.05, **p < 0.001. D. Schematic of the experimental procedure for treating 24-month-old mice with pyrintegrin. E. Representative immunostaining images showing the changes in K14, CD31, and PCNA expression in the dorsal skin of mice after pyrintegrin treatment, n = 3. F. Volcano plot showing the number of differentially expressed genes between the pyrintegrin group and the control group in RNA-seq. G. Bar graph showing the KEGG enrichment analysis terms of upregulated genes in the pyrintegrin group. H. Heatmap showing the changes in expression of angiogenesis-related genes in RNA-seq, n = 3 per group. I. Schematic illustrating the effects of pyrintegrin on vascular endothelial cells and epidermis in old mice.

To elucidate the role of integrin receptors in endothelial cell biology in aged skin, we analyzed scRNA-seq datasets from the dorsal skin of young (2-month-old) and aged (24-month-old) mice. After preprocessing and dimensionality reduction, we identified 20,318 cells (Figure S3A-B) and observed a significant age-related reduction in the proportion of EC (Figure S3C), consistent with the decline previously reported in aged human skin (Figure 1B). CellChat analysis of intercellular communication revealed a marked weakening of fibroblast- vascular endothelial cell (FB-VEC) interactions mediated by collagen-integrin signaling in aged murine skin (Figure 3A), also mirroring findings from human skin analyses. ScRNA-seq profiling demonstrated conserved but age-diminished expression of Itga9 and Itgb1 in mouse VECs (Figure 3B), a pattern further validated by RT-qPCR in young versus aged skin tissues (Figure 3C).

To functionally assess the impact of integrin receptor activation in aged skin, we administered pyrintegrin, a small-molecule activator of ITGB1, to 24-month-old mice (Figure 3D). Immunostaining revealed a significant increase in CD31^+^ endothelial cells and epidermal thickening in treated animals compared to controls (Figure 3E, Figure S3D).

Enhanced proliferative activity was evidenced by elevated BrdU incorporation (Figure S3E), while increased Collagen III fluorescence intensity suggested partial rejuvenation of the dermal matrix (Figure S3E). Following treatment with an ITGA9-specific activator (SU6656) on aged mouse dorsal skin, both proliferating cells in the epidermis and CD31-positive cells in the dermis exhibited significant increases in quantity (Figure S3F), These results suggest that enhancing ITGB1 or ITGA9 function in aged mouse skin could serve as an effective strategy to promote tissue rejuvenation. We then performed bulk RNA-seq analysis of the pyrintegrin-treated skin and the transcriptomic profiling identified 333 upregulated and 603 downregulated genes (Figures 3F, Figure S3G). KEGG enrichment analysis of upregulated genes highlighted angiogenesis as the top-ranked pathway (Figure 3G), with key mediators including Vegfa, Vcan, Pdgfra, Cxcl16, and Jag2 (Figure 3H). Reactome pathway analysis further revealed enrichment in developmental programs, immune regulation, cell cycle progression, and ECM reorganization (Figure S3H), suggesting multi-modal mechanisms through which integrin activation may counteract skin aging. Collectively, these findings demonstrate that age-related attenuation of collagen-integrin signaling contributes to vascular rarefaction in skin aging.

### Exploring integrin receptor modulation to rejuvenate aged skin cells through the skin organoid model

**Figure 4:**
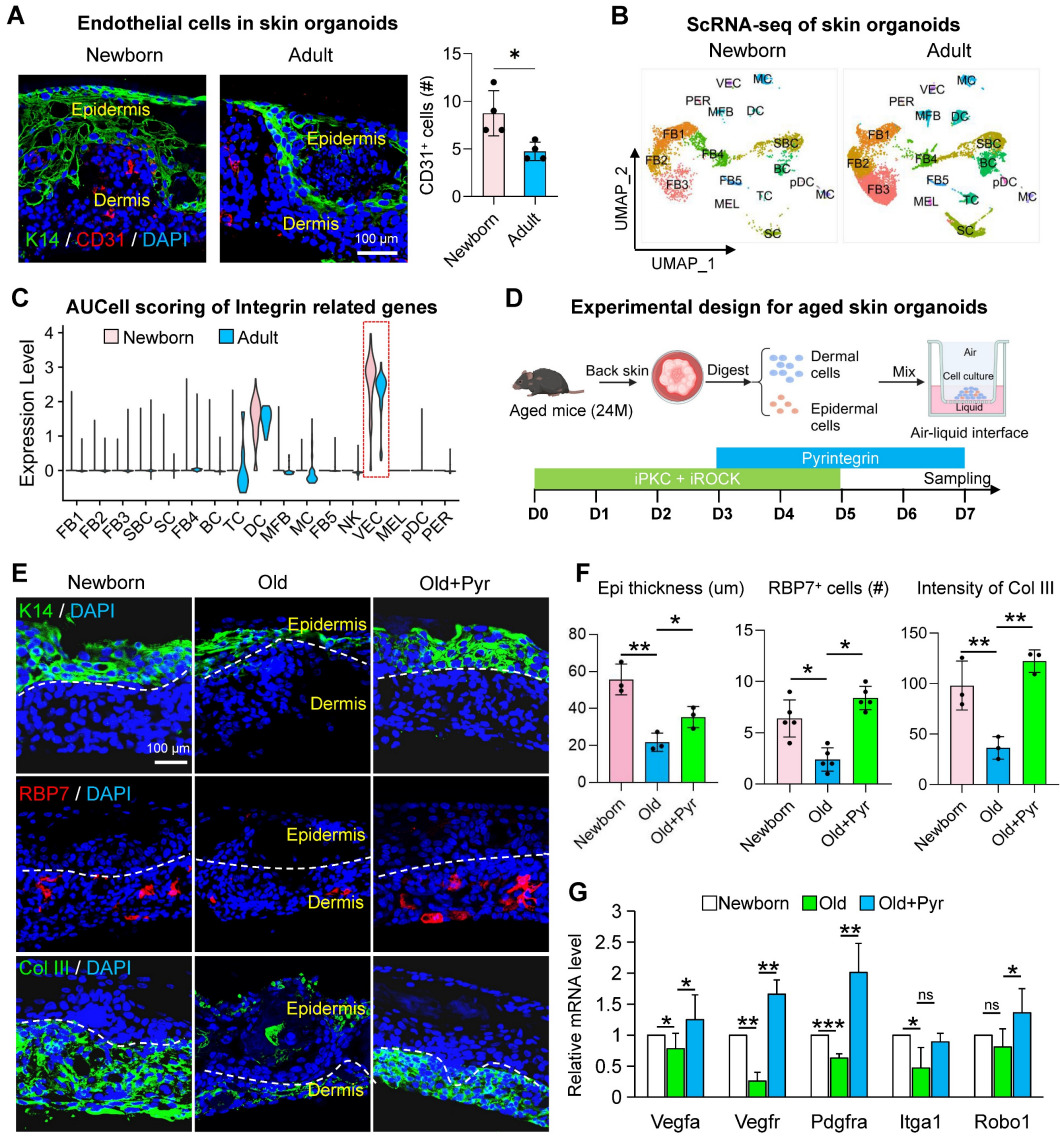
** Integrin signaling modulation restores youthful phenotypes in aged skin organoids.** A. Representative immunofluorescence images showing the expression of the endothelial cell marker CD31 in skin organoids derived from neonatal and adult mice. n = 3. B. UMAP plot showing the distribution of cell subclusters in scRNA-seq data from skin organoids derived from neonatal (P0) and adult mice (P60). C. Violin plot comparing the expression scores of integrin receptor genes related to biomechanics in skin organoids from neonatal and adult groups. D. Schematic illustration of the construction of skin organoids derived from the skin of aged mice. E. Representative immunofluorescence images comparing the expression of basal epidermal cell marker K14, endothelial cell marker RBP7, and dermal fibroblast marker Col III in Newborn, Old, and Old + Pyr groups. N = 3. F. Quantitative analysis of CD31-positive cells (left panel), RBP7-positive cells (middle panel), and fluorescence intensity of Col III (right panel) in Newborn, Old, and Old + Pyr groups. N = 4, *p < 0.05, **p < 0.01. G. RT-qPCR analysis comparing the relative expression levels of Vegfa, Vegfr, Pdgfra, Itga1, and Robo1 in Newborn, Old, and Old + Pyr groups. N = 3, *p < 0.05, **p < 0.01, ***p < 0.001, “ns” means no significance.

We have previously established skin organoids derived from newborn mice, which provide a robust platform for modeling skin development and intercellular interactions during skin disease progression 30, 31. However, the field of skin organoid research utilizing aged cells remains underexplored. Aged mouse skin organoids exhibit impaired self-organization into planar dermal and epidermal structures, accompanied by a marked reduction in vascular endothelial cell populations. To determine whether integrin receptor activation could enhance the self-organization capacity of aged skin organoids and mitigate their senescent phenotypes, we conducted a series of experiments. Using dorsal skin from 18-month-old C57 mice, we generated aged skin organoids and compared their structural organization to those derived from newborn mice. Immunostaining revealed a significant decline in vascular endothelial cell numbers in adult organoids relative to newborn controls (Figure 4A). To elucidate the relationship between reduced endothelial cell populations and integrin receptor gene expression, we analyzed scRNA-seq data from newborn and adult mouse skin organoids. Integrated clustering and dimensionality reduction identified 17 distinct cell clusters (Figure 4B, Figure S4A). Integrin receptor gene scoring demonstrated a pronounced reduction in expression within vascular endothelial cells of aged mice (Figure 4C), consistent with patterns observed in both human and murine skin. Co-expression analysis further revealed that the co-expression of Itga9 and Itgb1 in endothelial cells was significantly diminished in aged organoids compared to young counterparts (Figure S4B). Single-cell differential enrichment analysis indicated that in young mouse skin, cells co-expressing Itga9 and Itgb1 were predominantly enriched in pathways associated with cell division and the cell cycle. In contrast, aged mice exhibited enrichment in pathways related to the negative regulation of the TCF signaling pathway, potentially contributing to the reduced vascular endothelial cell numbers observed in aged organoids (Figure S4C). Notably, our senescence-modeling skin organoids demonstrated coordinated downregulation across multiple gene modules, including ECM biosynthesis, SASP components, and epidermal morphogenesis regulators (Figure S4D). This molecular signature aligns with the transcriptional landscape of aged murine skin, validating the organoid system's capacity to faithfully recapitulate key aspects of cutaneous aging pathophysiology. While our skin organoid model does not incorporate additional systemic factors such as inflammation, hormonal fluctuations, or environmental stressors, it successfully recapitulates key aging phenotypes of the skin, including diminished dermal collagen synthesis, impaired epidermal stratification, and activation of the SASP.

To mechanistically interrogate the collagen-integrin axis in cutaneous senescence, we implemented a combinatorial pharmacological strategy in skin organoids. Building upon the established protein kinase C (PKC) and Rho-associated kinase (ROCK) inhibitor protocol, systemic potentiation of integrin signaling was achieved through pyrintegrin supplementation. Following a sustained 7-day culture regimen (Figure 4D). This intervention resulted in a significant increase in the number of K14 layers and RBP7-positive cells within aged organoids (Figure 4E-F). To further investigate the impact of pyrintegrin on angiogenesis-related gene expression, we performed an RT-qPCR analysis. Notably, the expression of Vegfa, Vegfr, Pdgfra, and Robo1 was significantly upregulated following pyrintegrin supplementation (Figure 4G), suggesting pyrintegrin orchestrates an angiogenic program in aged skin organoids.

### Vascular endothelial cells modulate skin self-renewal through paracrine signaling during aging

**Figure 5:**
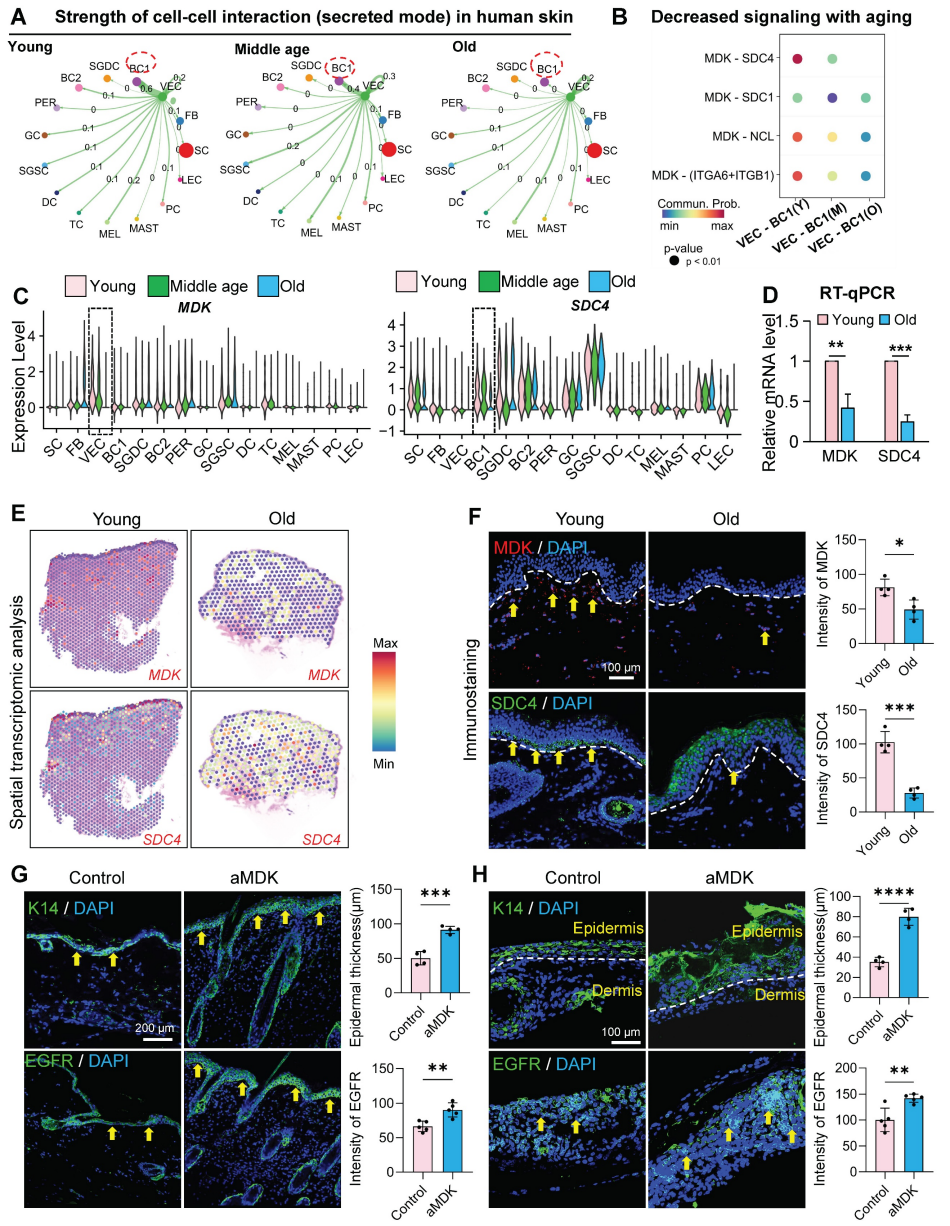
** Endothelial-derived paracrine factors regulate epidermal regenerative capacity in aging skin.** A. Circle plot comparing the interactions of VEC as signal senders with other cell clusters across young, middle-age, and old human buttock skin. B. Bubble plot showing the changes in interaction strength between VEC and BC based on MK (midkine) signaling with increasing age. N = 4, **p < 0.01, ***p < 0.001. C. Violin plot showing the expression changes of MDK and SDC4 in scRNA-seq data of human facial skin. D. RT-qPCR analysis comparing the relative expression levels of MDK and SDC4 in human skin. N = 3, **p < 0.01, ***p < 0.001. E. ImageFeaturePlot showing the expression changes of MDK and SDC4 in spatial transcriptomics data of young and aged human skin. F. Left panel: Representative immunostaining images showing the expression of MDK and SDC4 in young and aged human skin. Right panel: Quantitative analysis of relative fluorescence intensity of MDK and SDC4. N = 3, *p < 0.05, ***p < 0.001. G. Left panel: Representative immunofluorescence images comparing the expression levels of K14 and EGFR in control and aMDK (treated by MDK activator) groups of mice. Right panel: Quantitative analysis of epidermal thickness and relative fluorescence intensity of EGFR in control and aMDK groups. N = 3, **p < 0.01, ***p < 0.001. H. Left panel: Representative immunofluorescence images comparing the expression levels of K14 and EGFR in skin organoids from control and aMDK groups. Right panel: Quantitative analysis of epidermal thickness and relative fluorescence intensity of EGFR in skin organoids from control and aMDK groups. N = 3, **p < 0.01, ****p < 0.0001.

Vascular endothelial cells play a multifaceted role in skin biology, driving angiogenesis and regulating epidermal cell self-renewal and dermal fibroblast collagen synthesis through paracrine signaling. To investigate whether VECs influence skin self-renewal during aging, we employed CellChat to analyze cell-cell communication networks in young, middle, and aged skin. Our analysis revealed a progressive decline in interactions between ECs and basal cells with advancing age (Figure 5A). Notably, the secretion of Midkine (MK) signals from ECs was markedly reduced in aged skin (Figure S5A), and the MDK-SDC4 signaling axis was significantly diminished in the aged group (Figure 5B). Further examination using violin plots demonstrated that MDK is predominantly expressed in VECs, with its expression declining with age. Similarly, SDC4, which is also expressed in the epidermis, exhibited the most pronounced reduction in VECs during aging (Figure 5C). Similarly, the MK signaling-mediated interaction between VEC and BC in eyelid skin showed progressive decline during aging (Figures S5B-C). To validate these findings in human skin, we performed RT-qPCR and observed a significant decrease in MDK and SDC4 expression in aged human skin samples (Figure 5D). Furthermore, we analyzed Mdk and Sdc4 expression in dorsal skin tissues across age groups. Mdk levels exhibited gradual decline starting at 12 months of age, while Sdc4 expression decreased from 9 months onward (Figure S5D). Spatial transcriptomic analysis of human skin further confirmed the age-related decline in MDK and SDC4 expression (Figure 5E), a result corroborated by immunostaining (Figure 5F).

To explore the functional impact of MDK supplementation on aged skin, we administered recombinant MDK protein to aged mice (Figure 5G) and aged mouse skin organoids (Figure 5H) and assessed changes in the epidermal layer. Treatment with MDK resulted in a significant increase in the number of K14-positive cell layers and enhanced expression of the epidermal growth factor receptor (EGFR) (Figure 5G-H), indicating improved epidermal self-renewal capacity.

### Identification of MDK-SDC4 downstream signaling pathways

**Figure 6:**
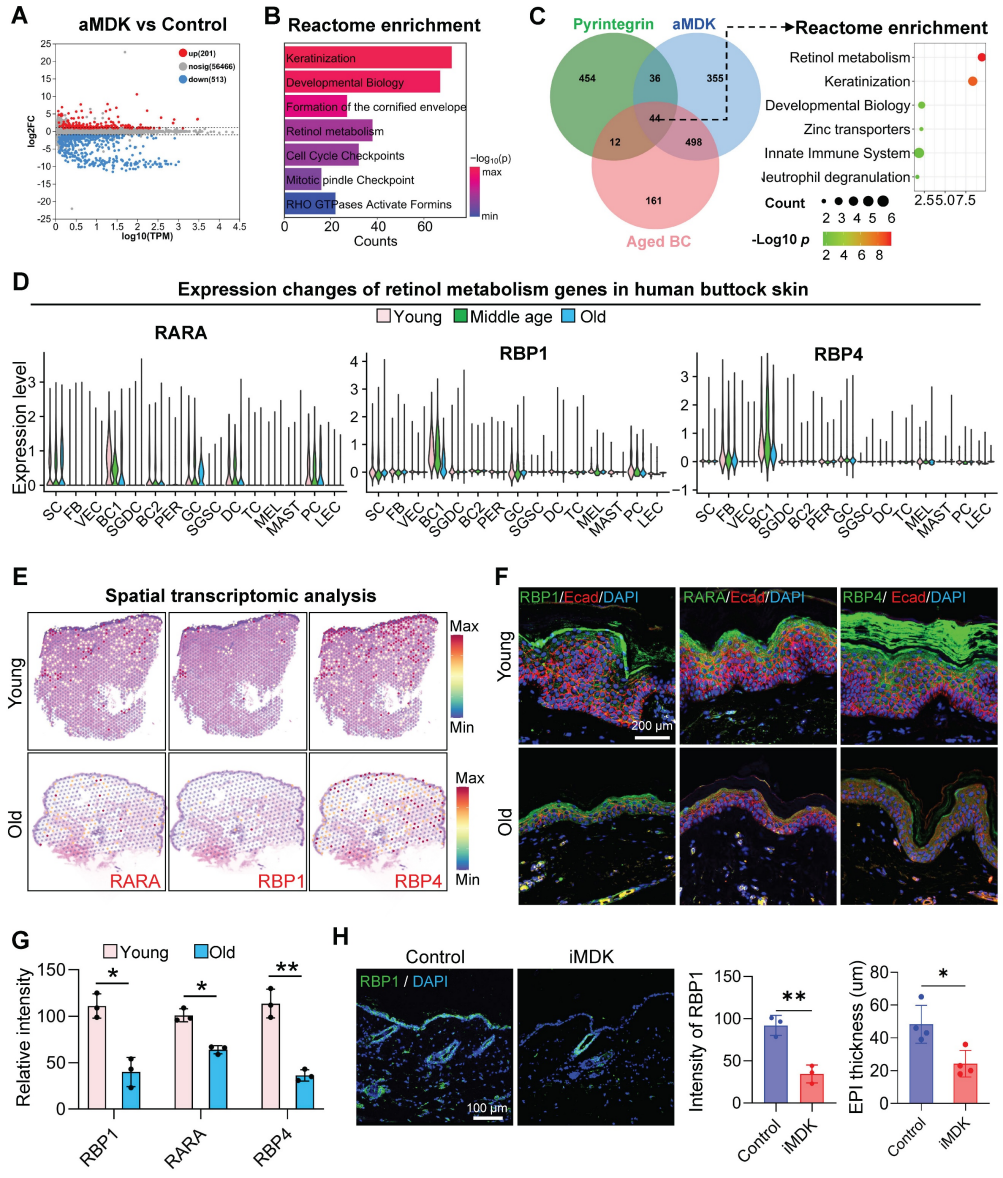
** Deciphering the Mdk-Sdc4 signaling axis in cutaneous homeostasis.** A. Volcano plot showing differentially expressed genes between the aMDK group and the control group in RNA-seq analysis. B. Bar graph showing Reactome enrichment analysis terms of upregulated genes in the aMDK group. C. Left panel: Venn diagram showing the overlap of upregulated genes in the aMDK group, upregulated genes in the Pyr group, and downregulated genes in aged basal cells. Right panel: Reactome enrichment analysis of intersection genes of three groups. D. Violin plot showing the expression changes of retinol metabolism genes RARA, RBP1, and RBP4 in scRNA-seq data of human facial skin, n = 3, **p < 0.01, ***p < 0.0001. E. ImageFeaturePlot showing the expression changes of RARA, RBP1, and RBP4 in spatial transcriptomics data of young and aged human skin. F. Representative immunofluorescence images showing the expression of RARA, RBP1, and RBP4 in young and aged human skin, n = 3. G. Quantitative analysis of relative fluorescence intensity of MDK and SDC4. N = 3, *p < 0.05, **p < 0.01. H. Left panel: Representative immunofluorescence images showing changes in RBP1 expression in the control and iMDK groups. Right panel: Quantitative analysis of relative fluorescence intensity of RBP1 in the control and iMDK groups. N = 3, *p < 0.05, **p < 0.01.

To elucidate the downstream signaling pathways of MDK-SDC4, we performed RNA-seq on skin samples from aged mice injected with an MDK activator (aMDK) and identified 201 upregulated and 513 downregulated genes in the aMDK group compared to the control (Figure 6A). Reactome enrichment analysis of the upregulated genes revealed significant enrichment in pathways related to keratinization, developmental biology, formation of the cornified envelope, and retinol metabolism (Figure 6B).

We then conducted a comparative enrichment analysis of biological processes in samples treated with pyrintegrin and MDK activators. Both treatments exhibited enrichment in retinol metabolism pathways (Figure S6A). To further investigate metabolic signals in the epidermis, we performed a Venn diagram analysis of genes upregulated by pyrintegrin and MDK activators and downregulated in aged epidermal cells. Intriguingly, this analysis identified 44 overlapping genes. Enrichment analysis demonstrated that these genes were closely associated with retinol metabolism (Figure 6C). Retinol metabolites, such as retinyl ester and retinoic acid, are known to promote the self-renewal of epidermal basal cells and maintain skin barrier function 32.

Given the observed increase in retinol metabolism following treatment with mechanical receptor activators and MDK, we hypothesized that the MDK-SDC4 signaling pathway between endothelial cells and the epidermis modulates retinol metabolism in epidermal cells. To validate age-related changes in retinol metabolism, we analyzed scRNA-seq data from human eyelid skin, focusing on key retinol metabolism genes (RARA, RBP1, and RBP4). These genes were highly expressed in epidermal basal cells but declined significantly with age (Figure 6D). Spatial transcriptomic analysis (Figure 6E) and immunostaining (Figure 6F-G) further confirmed that the expression of these retinol metabolism proteins was predominantly localized in the epidermal layer and decreased markedly with aging.

To assess the impact of MDK inhibition on retinol metabolism, we treated the dorsal skin of young mice with an MDK inhibitor. This resulted in a significant reduction in the expression of the retinol metabolism-related protein RBP1 in epidermal cell clusters (Figure 6H). Conversely, treatment of aged mice with an MDK activator led to a marked increase in the expression of retinol metabolism-related genes (Figure S6B). Immunostaining further demonstrated that the expression of RARA, a key gene in retinol metabolism, was significantly elevated in the epidermal cells of aged mice following MDK activation (Figure S6C). Additionally, the number of PCNA-positive cells, a marker of cell proliferation, increased notably in the epidermis (Figure S6C). These findings suggest that MDK enhances retinol metabolism in epidermal cells and promotes their self-renewal capacity.

### Functional validation of MDK-SDC4-mediated interaction between EC and BC promotes epidermal self-renewal via retinol metabolism

**Figure 7:**
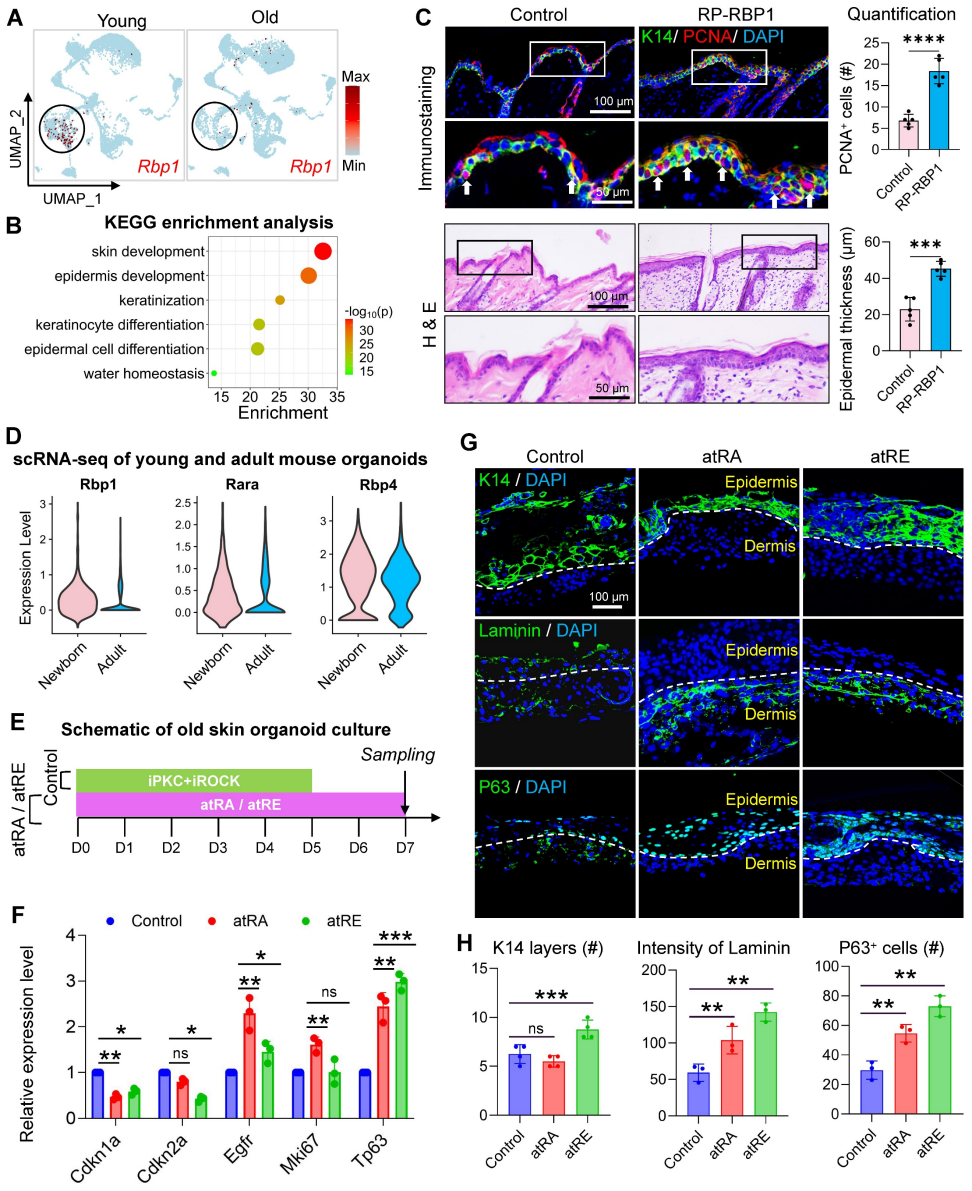
** Mdk-Sdc4 axis orchestrates epidermal regeneration through retinoid-mediated endothelial-basal cell crosstalk.** A. FeaturePlot showing the expression changes of Rbp1 in scRNA-seq data from young and aged mouse skin. B. KEGG enrichment analysis highlighting the signaling pathways specifically enriched in Rbp1-positive cells in mouse skin. C. Upper panel: Representative immunofluorescence images showing the expression of K14 and PCNA in RP-RBP1 and control groups (left), and quantitative analysis of PCNA-positive cell numbers (right). N = 3, ****p < 0.0001. Lower panel: Representative H&E staining images showing changes in the epidermis of RP-RBP1 and control groups (left), and quantitative analysis of epidermal thickness. N = 3, ***p < 0.001. D. Violin plot showing the expression levels of Rbp1, Rara, and Rbp4 in epidermal cell clusters of skin organoids derived from neonatal and aged mice. E. Schematic diagram illustrating the reprogramming of aged mouse skin organoids using all-trans retinoic acid (atRA) and all-trans retinol (atRE). F. RT-qPCR analysis comparing the expression levels of senescence-associated markers Cdkn1a and Cdkn2a, as well as epidermal growth-related genes Egfr, Mki67, and Tp63 in control, atRA, and atRE groups, n = 3, *p < 0.05, **p < 0.01, ***p < 0.001, “ns” means no significance. G. Representative immunofluorescence images showing the expression of K14, Laminin, and P63 in control, atRA, and atRE groups. H. Quantitative analysis of the number of K14-positive cell layers, Laminin signal intensity, and P63-positive cell counts, n = 3, **p < 0.01, ***p < 0.001, “ns” means no significance.

Our above analysis suggests that the MDK-SDC4-based interaction between VEC and BC may help maintain the self-renewal capacity of young skin, potentially through its downstream regulation of retinol metabolism. Notably, the expression of the retinol-binding protein gene Rbp1 was significantly increased following MDK activator treatment. Interestingly, compared to young mice, the expression of RBP1 in epidermal cells was markedly reduced in aged mouse skin samples (Figure 7A).

To further explore the role of RBP1, we performed gene expression enrichment analysis on cells with high and low RBP1 expression. The results revealed that cells with high RBP1 expression were enriched in pathways related to skin development and epidermal differentiation, suggesting a critical role for RBP1 in epidermal self-renewal (Figure 7B). Despite its potential importance, the role of RBP1 in maintaining skin youthfulness remains poorly understood. To investigate if RBP1 can promote skin rejuvenation in aged mice, we administered recombinant RBP1 protein to the aged mouse skin. This treatment resulted in epidermal thickening and an increase in the number of PCNA-positive cells (Figure 7C). RT-qPCR analysis further demonstrated that RBP1 treatment significantly reduced the expression of senescence associated markers (Cdkn1a and Cdkn2a) and increased the expression of key epidermal development genes (Egfr and Tp63), indicating that RBP1 promotes skin rejuvenation in aged mice (Figure S7A). These findings suggest that retinol-binding proteins may enhance the ability of cells in the epidermal microenvironment to bind retinol, which is subsequently metabolized into retinyl esters and retinoic acid.

To validate the impact of retinol metabolism on aged skin organoids, we compared the expression of Rbp1, Rara, and Rbp4 in newborn and adult skin using scRNA-seq analysis. The results showed that the expression of all three genes was significantly reduced in adult skin, with Rbp1 exhibiting the lowest relative expression (Figure 7D), consistent with trends observed in human skin samples. Given that retinol metabolites, such as retinyl ester and retinoic acid, may play a key role in reversing skin aging, we supplemented aged mouse skin-derived organoids with all-trans retinol acid (atRa) and all-trans retinyl ester (atRE) (Figure 7E). Treatment with atRE and atRA significantly reduced the expression of Cdkn1a and Cdkn2a and increased the expression of Egfr and Tp63. While Mki67 expression did not change significantly in the atRE group (Figure 7F), immunostaining revealed that atRA and atRE treatments stabilized the basement membrane structure between the dermal and epidermal layers of aged skin organoids. Additionally, the number of P63-positive epidermal stem cells increased, and epidermal cells formed a stable epidermal layer (Figure 7G-H). Finally, we validated the rejuvenating effects of retinoid metabolites in aged mice (Figure S7B). Three retinoid compounds (ROL, HPR, RP) were administered via alternate-day intradermal injections to 24-month-old mice. Compared with vehicle controls, all retinoid-treated groups exhibited significant increases in epidermal thickness and proliferating cell numbers. Notably, Col III expression was markedly upregulated in treated groups, indicative of senescent phenotype reversal in aged skin (Figure S7C-D). These results suggest that retinyl esters and retinoic acid are key mediators of the MDK-SDC4-RBP1 axis, ultimately promoting epidermal self-renewal. This further supports the notion that EC-mediated regulation of BC represents a promising new direction for anti-aging interventions in the skin.

## Discussion

Skin aging is characterized by the continuous deterioration of its structure and functionality due to the accumulation of both intrinsic and extrinsic molecular changes. These changes manifest as wrinkles, dryness, compromised barrier integrity, and epidermal thinning 2, whereas how different types of cells interact to transduce mechano-chemical signaling to drive skin aging remains largely unknown. By analyzing scRNA-seq data from human eyelid skin across different age groups, we identified significant age-related declines in the proportion and proliferative capacity of vascular VECs, coupled with alterations in ECM organization and ECM-receptor interactions, particularly between fibroblasts and VECs. Aging FBs exhibited a marked reduction in ECM-related gene expression, leading to increased matrix stiffness, impaired collagen-integrin signaling, and stagnation of VEC proliferation. Notably, supplementation with type I collagen restored VEC numbers, highlighting the critical role of ECM composition in sustaining VEC function. Furthermore, we observed reduced angiogenic capacities in aging fibroblasts. Our findings also reveal that MDK-SDC4 signaling between VECs and basal epidermal cells declines with age, disrupting epidermal retinol metabolism and impairing epidermal renewal. Importantly, this impairment is reversible through exogenous retinol supplementation, highlighting potential therapeutic targets, such as collagen-integrin signaling and MDK-SDC4 pathways, to counteract age-related skin changes.

Dermal fibroblasts are essential in maintaining skin elasticity and strength by supplying the dermis with abundant type I and III collagen, which also acts as scaffolding for skin appendages and other constituents such as dermal microvasculature. However, during aging, fibroblast activity wanes and collagen degradation increases, resulting in skin laxity and wrinkle formation 17. Leading theories often suggest vascular remodeling as a consequence of its tissue microenvironment 33. Surprisingly, we discovered a significant age-dependent decline in the expression of growth-related genes in VECs, a trend more pronounced than those found in fibroblasts of the epidermis and dermis. This suggests that dermal VEC aging may precede that of other skin cell types instead of being reactionary to the alterations of its microenvironment 34, 35. Indeed, aging endothelial cells exhibit reduced angiogenic capacity, diminished proliferation, and increased secretion of pro-inflammatory cytokines, which collectively disrupt vascular homeostasis and tissue maintenance 36, 37. This pro-inflammatory and pro-fibrotic microenvironment created by aging VECs exacerbates cellular aging in neighboring fibroblasts and stromal cells, further accelerating tissue degeneration, fibrosis, and loss of elasticity 37. Consequently, dysfunctional endothelial cells facilitate ECM remodeling by increasing collagen cross-linking and stiffening due to the accumulation of advanced glycation end products (AGEs) and reduced matrix turnover, further impairing tissue repair and regeneration 17, 38.

On the other hand, endothelial cells are particularly vulnerable to age-related damage due to their sensitivity to biomechanical forces and biochemical changes in the perivascular ECM 36. Dermal microvasculature can sense the ever-changing stiffness in the perivascular space and initiate self-remodeling as a response. The stiffening of the ECM alters cell-ECM interactions, particularly integrin-mediated mechano-transduction, as observed in our study, which is essential for maintaining VEC homeostasis and angiogenesis 39. Moreover, dermal vascular VECs can sense and readily react to subtle changes in the stiffness of the perivascular microenvironment. For instance, moderate dermal ECM stiffness increases the expression of APJ, a force-sensing molecule on vascular VECs, which induces angiogenesis and vascular maturation. However, excessive dermal ECM stiffness is detrimental to this process as it dramatically downregulates APJ expression, leading to a recession of the dermal microvascular network. Thus, as skin ages, this cascade of endothelial-driven events induces continuous remodeling of the ECM and increases the perivascular tensile modulus, which is subsequently sensed by vascular VECs and causes further deterioration of the dermal vascular network 40.

One effective way for aging vascular VECs to trigger systemic tissue aging is through paracrine signaling via various secreted factors, which are critical to vascular dysfunction and metabolic dysregulation. Nevertheless, both vascular VECs and keratinocytes are crucial for the structural and functional integrity of the skin, with their interactions supporting wound healing and physiological regulation 41. Targeting these cells may offer promising strategies for alleviating aging-related systemic metabolic disorders. In the present study, we reveal a key pair of signaling molecules, MDK and its receptor Syndecan-4 (SDC4), that hold the potential to serve as therapeutic targets. This is inspired by our observation of a significant reduction in the secretion of MDK, a heparin-binding growth factor, by aging VECs, alongside a decline in the expression of its receptor, SDC4, in basal epidermal cells. MDK, a molecule known for its role in leukocyte migration and tumor progression 42, has emerged as a critical mediator of epidermal self-renewal and repair. Notably, MDK activation significantly increases the secretion of EGF by keratinocytes, which is crucial for supporting VEC growth, angiogenesis, and skin homeostasis 43-45. This suggests a reciprocal regulatory mechanism between VECs and basal epidermal cells within the skin microenvironment, wherein MDK acts as a central signaling hub connecting vascular and epidermal compartments to maintain tissue integrity.

More importantly, our results suggest that MDK stimulation activates SDC4-mediated retinoid metabolic signaling in basal epidermal cells, thereby promoting epidermal renewal. Retinoids, including retinol and its active metabolites, are well-established regulators of gene expression involved in skin cell proliferation, differentiation, and barrier function 46. Retinol, a fat-soluble vitamin, has been shown to enhance keratinocyte and VEC proliferation in aged skin, restoring the epidermal-dermal balance and exerting potent anti-aging effects 47. Through signaling via retinoic acid receptors (RARs), retinoids downregulate keratinocyte differentiation, maintaining a more immature and regenerative epidermal state 48. Furthermore, studies confirm that topical retinol application improves epidermal barrier function, enhances keratinocyte proliferation, and promotes overall skin health in both in vitro and in vivo models 49-51. Collectively, these findings underscore the significance of the MDK-SDC4 axis in mediating not only vascular-epidermal crosstalk but also in regulating retinoid signaling pathways that are vital for epidermal regeneration and anti-aging interventions.

In summary, our study highlights the critical role of ECM integrity, intercellular communication, and mechanochemical signaling in skin aging. We identified key pathways, such as collagen-integrin signaling and MDK-SDC4-mediated retinoid metabolism, as central regulators of endothelial and epidermal cell function with age. These findings not only deepen our understanding of the cellular and molecular mechanisms underlying skin aging but also reveal promising therapeutic targets for mitigating age-related skin changes. Future research should further explore the therapeutic potential of MDK and integrin receptor activators in both preclinical and clinical settings. Additionally, the development of advanced skin organoid models using aged cells will provide valuable tools for studying the underlying molecular mechanisms and evaluating anti-aging interventions. Expanding upon the interplay between ECM composition, mechanical forces, and intercellular signaling may uncover new strategies to preserve skin homeostasis and rejuvenate aged skin. Certainly, the specific mechanisms by which vascular endothelial cell dysregulation contributes to the aging of the skin microenvironment require further elucidation through large-scale, long-term clinical studies. Moreover, the interplay between additional cell populations within the skin microenvironment, particularly immune cells, adipocytes, and other stromal components, warrants systematic investigation to fully delineate their collaborative roles in age-related tissue remodeling.

## Materials and Methods

### Human skin samples

Young (average 18 years old) and aged (average 83 years old) human skin samples were obtained from the Three Gorges Hospital. Normal skin tissues were derived from surgical excision of lipomas and were collected at the First Affiliated Hospital of Chongqing University (Chongqing, China). All experimental protocols involving human tissues were approved by the Ethics Committee of Chongqing University.

### Mice and treatment

C57BL/6J mice (Charles River Laboratories, Beijing) were stratified into two cohorts: young adult (2-month-old) and aged (24-month-old) groups. Following dorsal hair removal with electric clippers, mice received daily subcutaneous injections (50 μL/mouse) of the following agents for 7 consecutive days: Pyrintegrin (50 μM in PBS; MedChemExpress, HY-13306); SU6656 (40 μM in PBS; MedChemExpress, HY-B0789); Recombinant RBP1 protein (100 μg/mL in PBS; MedChemExpress, HY-P71093); MDK signaling inhibitor (20 μM in PBS; MedChemExpress, HY-110171); Three retinoid metabolites (Rol, RP, and HPR) were sourced from TargetMol and applied at a concentration of 50 μM each; Control groups received equivalent volumes of PBS vehicle. On day 8 post-initial treatment, mice were euthanized via CO_2_ asphyxiation followed by cervical dislocation. Full-thickness dorsal skin specimens were immediately fixed in 4 % paraformaldehyde (Servicebio, G1101) for 24 hours at 4 ℃ prior to paraffin embedding and sectioning.

### Single cell RNA-seq analysis

ScRNA-seq data from human buttock skin across young, middle-aged, and aged cohorts were sourced from the Gene Expression Omnibus (GEO accession: GSE274955). Age-stratified scRNA-seq datasets of human eyelid skin were obtained through the Genome Sequence Archive (GSA project ID: HRA000395). Additional scRNA-seq datasets were obtained from the GEO database, including: (1) young and aged human groin skin samples (GSE130973), (2) dorsal skin samples from young and aged mice (GSE132042), and (3) skin organoid data previously deposited by our group (GSE215980). Following data acquisition, raw gene expression matrices, barcode information, and feature annotations were processed using Seurat (v.3.0.053) for quality control, dimensionality reduction (UMAP/t-SNE), cell cluster identification, and differential gene expression analysis. The quality control process was performed using Seurat. For quality control, features detected in less than 5 cells, and cells with mitochondrial genes greater than 5 % were removed. Single cells with less than 400 UMIs or with more than 5000 UMIs or with more than 20 % mitochondrion-derived UMI counts were considered low-quality cells and removed. Doublet Finder package (version2.0.3) was used for calculating doublets. The mean-variance-normalized bimodality coefficient (BCMVN) of each sample was used to calculate the neighborhood size (pK), and the number of artificial doublets (pN) was set to 0.25. Batch effects among the patients were eliminated using the Harmony. The FindAllMarkers function was used to list the markers of each cell cluster. The major cell types were then recognized based on the markers obtained from the CellMarker database and previous studies.

### CellChat analysis

To assess cell-to-cell interactions between different cell types in young and aged human skin we used CellChat V.1.6.114, which is based on the expression of known ligand-receptor pairs. The cell-cell interactions between different cell types in the DRG dataset were evaluated using CellChat (Version 1.4.0, R package). CellChat takes gene expression data as user input to model the probability of cell-cell communication by integrating gene expression with the existing database consisting of known interactions between signaling ligands, receptors, and their cofactors. The significance of the communication probability is determined by assessing whether it is statistically higher between the known cell types than between randomly permuted groups of cells.

### Bulk RNA-seq

For bulk RNA-seq, cDNA libraries were prepared according to the manufacturer's instructions for the TruSeq Stranded mRNA Sample Prep Kit for the skin tissue (Illumina, San Diego, CA). The concentration and size distribution of the completed libraries were determined using an Agilent Bioanalyzer DNA 1000 chip (Santa Clara, CA) and Qubit fluorometry (Invitrogen, Carlsbad, CA). We performed data format conversion on the raw data to compare the differentially expressed genes between the two groups. Firstly, we standardized expression levels by converting count values to CPM (Counts Per Million). The PCA plots and differential gene expression were analyzed with the R package DESeq2. Genes with log2 FC (fold change) > 1 and adjusted P value (by the Benjamini-Hochberg method) < 0.05 were considered DEGs. The DEGs were further divided into upregulated and downregulated genes according to log_2_FC for enrichment analysis. Finally, GO and KEGG databases were queried to understand the biological functions of each cluster. GO and KEGG enrichment was utilized for the annotation and pathway analysis of the differentially expressed genes.

### RT-qPCR

The total RNA of organoids was isolated using MagZol reagent (Magen, cat. R4801-01, China). Subsequently, complementary DNA (cDNA) synthesis was carried out by employing the HiScript Q RT Kit (Yeasen, cat.11141ES60, China) through reverse transcription. Following that, qRT-PCR was conducted on a QuantStudio 3 RT-PCR instrument (Thermo Fisher Scientific) using the SYBR Green Qpcr Master Mix with low ROX (Bimake, China) according to the manufacturer's guidelines. The obtained values were normalized using GAPDH as a reference gene, Information on all primers is provided in **Table S1**.

### Immunostaining

The method for embedding, sectioning, dewax, and rehydration of tissue specimens in this study faithfully adhered to the immunostaining protocols. Antigen retrieval was achieved via a 0.01 M citrate buffer (pH 6.0), and blocking was performed with a 2 % BSA solution, which was subsequently incubated in an oven at 37 °C for 2 h. A 1:200 dilution of the primary antibody was then applied to each sample, and incubated overnight at 4 °C for optimal reaction. Following completion of the primary antibody reaction, the specimens underwent thorough washing with PBST and PBS. Subsequently, a secondary antibody was prepared at a dilution of 1 : 500 and incubated for 2 h at 37 °C, information on all antibodies are provided in **Table S2**. After the removal of the secondary antibody, the nuclei were stained with DAPI. Comprehensive anti-fluorescence quenching agent treatment in slide mounting was eventually applied for optimal experimental fidelity.

### HE staining

Fresh human young skin, Middle-aged skin, and aged skin were fixed by 4 % PFA, routinely embedded in paraffin, and sliced into 7 mm sections by Leica slicer (Germany). The sections were then subjected to dewax twice with xylene for 15 min and hydrated using a gradient of ethanol (100 %, 95 %, 85 %, and 75 %) with each gradient lasting for 3 min. The samples were subsequently soaked in distilled water for 2 min, and the nuclei were stained with hematoxylin (Servicebio, cat. G1120, China) for 3 min, then rinsed with tap water. Next, the samples were differentiated for 3 min, and washed with tap water twice for 2 min. Then the samples were re-dyed with eosin for 2 min. Finally, the samples were penetrated by xylene and sealed with resinene (Servicebio, cat. G8590, China), and examined by light microscopy using the light microscope (Zeiss, Germany).

### Photonic crystal bio-force microscopy

Photonic crystal cellular force microscopy (PCCFM) is a technique that utilizes the diffraction of photonic crystals to visualize and quantitatively detect vertical cellular forces. This technique allows for real-time cellular force imaging at various scales, ranging from subcellular to tissue levels. Firstly, the frozen section of human young skin and aged skin with a thickness of 25 mm was positioned on a cantilever of an indentation platform. Subsequently, the section was gradually compressed in increments of 5 mm onto a photonic crystal hydrogel sensor, which was also 25 mm thick, and immersed in a PBS container. Simultaneously, a microscope and color camera were used to capture color images of the photonic crystals at different compression depths, resulting in an image stack. We performed high-speed imaging of vertical cellular forces over a wide field of view (1.3 mm × 1.0 mm), capturing approximately 20 frames per second. The photonic crystal hydrogel substrates (PCS) allowed the transformation of nanoscale deformations into perceptible color changes, enabling high-throughput, in situ visualization and quantification of minimal vertical cellular forces. Following the acquisition of PCCFM images, we extracted the hue values pixel by pixel from the obtained images and then determined the wavelengths corresponding to these hue values based on the curves in the images. Ultimately, the stiffness pattern of the human young skin and aged skin were calculated using the PCBF algorithm based on the obtained image stack. Finally, we further transformed the obtained deformation map for each pixel position into Young's modulus values using the equation.

### Mouse skin organoids culture

Full-thickness back skin was excised with ophthalmic scissors, and excess adipose tissue was scraped off. Skin pieces (about eight per 10 cm dish) were incubated overnight in 20 mL of 0.25 % trypsin. After digestion, the skin was physically torn into dermis and epidermis. The dermis was then treated with 0.5 % collagenase at 37 °C for 30 minutes. Following two rounds of filtration (1100 rpm for 5 min), the dissociated dermal and epidermal cells were mixed in a 9 : 1 ratio and placed in a 12-well insert culture system (JET). 700 µL of DMEM/F12 medium was added to the bottom of the insert, changing the medium daily. From day 4 to day 7, groups (young and aged) received treatments with recombinant proteins or small molecules, with samples collected on day 7 and fixed in 4 % PFA. On day 7 of culture, samples were collected for analysis.

### Statistical analysis

All experiments in this study were performed with a minimum of three replicates. We employed SPSS 19.0 software to conduct statistical analysis of the data from each group, which included one-way analysis of variance (ANOVA), multiple comparisons, and t-tests. We considered p value below 0.05 to be statistically significant.

## Supplementary Material

Supplementary figures and tables.

## Abbreviations

Extracellular matrix: ECM
Matrix metalloproteinases: MMPs
Single-cell RNA sequencing: scRNA-seq
Granulosum cells: GC
Basal cells: BC
Spinosum cells: SC
Pericytes: PC
Melanocytes: ME
Dendritic cells: DC
Hair follicle cells: HF
Endothelial cells: EC
Fibroblasts: FB
T cells: TC
Midkine: Mdk
Retinol-binding protein: Rbp
All-trans retinol acid: atRa
All-trans retinyl ester: atRE
Syndecan-4: SDC4
Quantitative reverse transcription PCR: RT-qPCR
Hematoxylin and eosin staining: H&E
RNA sequence of bulk samples: Bulk RNA-seq. Senescence-associated secretory phenotype, SASP
