# Supplementary material for: Endothelium-specific sensing of mechanical signals drives epidermal aging through coordinating retinoid metabolism
Source: Theranostics. 2025 Jun 12;15(14):7045–63. doi: 10.7150/thno.112299 (PMC12203922; doi:10.7150/thno.112299)
Supplement: Supplementary file 1 — Supplementary figures and tables. [file thnov15p7045s1.pdf]

*Supplementary Information*

# Endothelium-specific sensing of mechanical signals drives epidermal aging through coordinating retinoid metabolism

**Running title:** Mechano-sensitive endothelial cells drive skin aging

Xia Wu[1,#], Jiangming Zhong[2,#], Jingwei Jiang[3,#], Yi Zou[3,4], Dehuan Wang[3], Ziyan Chen[3], Mengyue Wang[3], Xinyu Shen[3], Zeming Li[3], Yang Xiao[3], Yuyan Yi[3], Fangqi Tang[3,4], Xiaoyu Long[3], Weiming Qiu[4], Qu Tang[5], Xiao Xiang[3], Xun Zhou[6,✉], Mingxing Lei[3,✉], Peng Shu[2,✉], Qiang Zhou[1,✉]

This file includes the following subsections:

- **Figure S1-7**
- **Table S1-2**

**Figure S1**

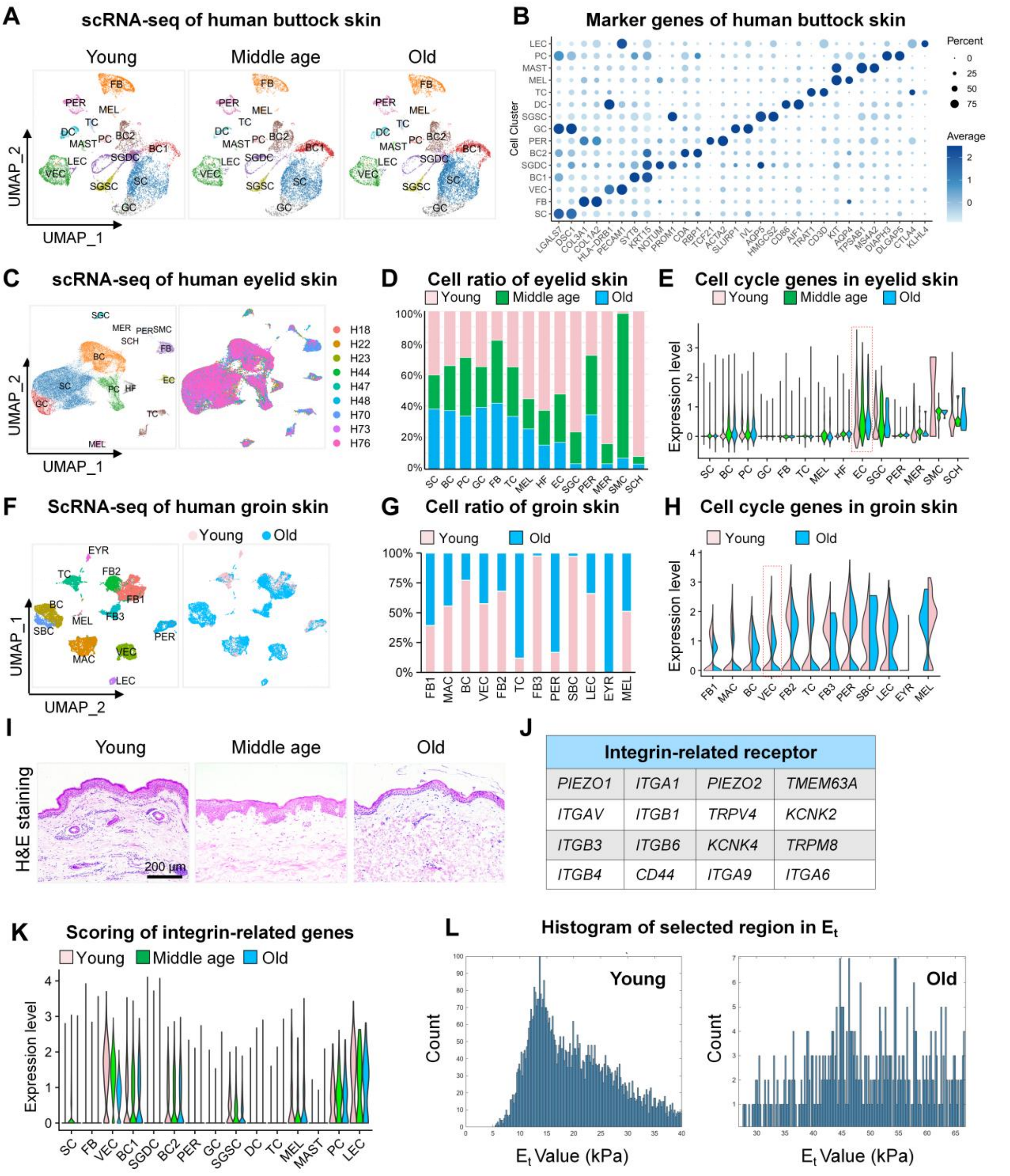


**Figure S1 Decreased expression of mechanical-related receptor genes in aging skin.**

A. UMAP plots illustrating the distribution of cellular populations in young, middle-aged, and aged human buttock skin.

B. Bubble plot displaying marker genes for each cell population in human buttock skin, with dot size and color intensity representing expression levels.

C. UMAP plots illustrating the distribution of cellular populations in young (18, 22,

23 years old), middle-aged (44, 47, 48 years old), and aged (70, 73, 76 years old) human eyelid skin.

D. Comparative analysis of cellular subset proportions in young, middle-aged, and old eyelid skin.

E. AUCell-based gene set enrichment scores for proliferation-associated genes across cellular clusters of human eyelid skin.

F. UMAP plots illustrating the distribution of cellular populations in young and aged human groin skin.

G. Comparative analysis of cellular subset proportions in young and old groin skin.

H. AUCell-based gene set enrichment scores for proliferation-associated genes across cellular clusters of human groin skin.

I. Representative H&E staining images depicting morphological changes in young, middle-aged, and aged skin, n = 3.

J. Gene set associated with matrix mechanics-related cell membrane receptors.

K. AUCell scores comparing the activity of matrix mechanics receptor-related genes across young, middle-aged, and aged skin groups.

L. Quantitative analysis of Young's modulus distribution across all pixels in young and aged skin, n = 3.

## Figure S2

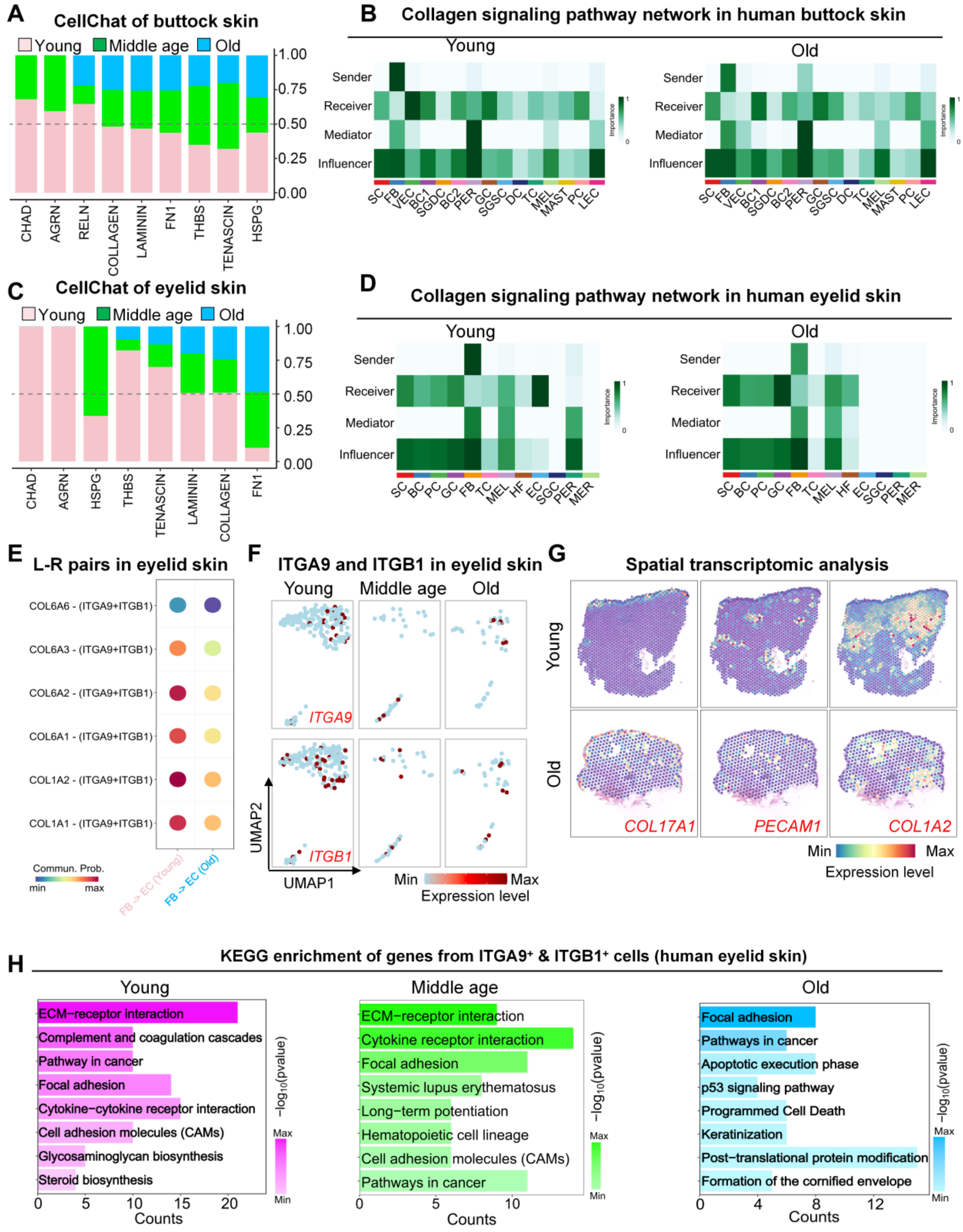


**Figure S2 CellChat analysis demonstrates enhanced Collagen-Integrin signal between FB and EC.**

A. Comparison of interaction strengths between FB and VEC mediated by the ECM-receptor signaling pathway in young, middle-aged, and aged buttock skin.

B. Importance analysis of senders, receivers, mediators, and influencers in the

collagen signaling pathway in young and aged buttock skin.

C. Comparison of interaction strengths between FB and VEC mediated by the ECM-receptor signaling pathway in young, middle-aged, and aged eyelid skin.

D. Importance analysis of senders, receivers, mediators, and influencers in the collagen signaling pathway in young and aged eyelid skin.

E. Dot plot illustrating differential Collagen-mediated interaction intensities between FB and VEC in young versus aged human eyelid skin.

F. Feature plots showing the expression of ITGA9 and ITGB1 in young, middle-aged, and aged eyelid skin.

G. ImageFeaturePlot visualization of spatial transcriptomic data showing expression of basal cell marker COL17A1, endothelial cell marker PECAM1, and fibroblast marker COL1A2 in young and aged human skin.

H. KEGG pathway enrichment analysis of highly expressed genes in ITGA9$^+$ and ITGB1$^+$ VECs from young (right panel), middle age (middle panel) and aged (lower panel) eyelid skin.

## Figure S3

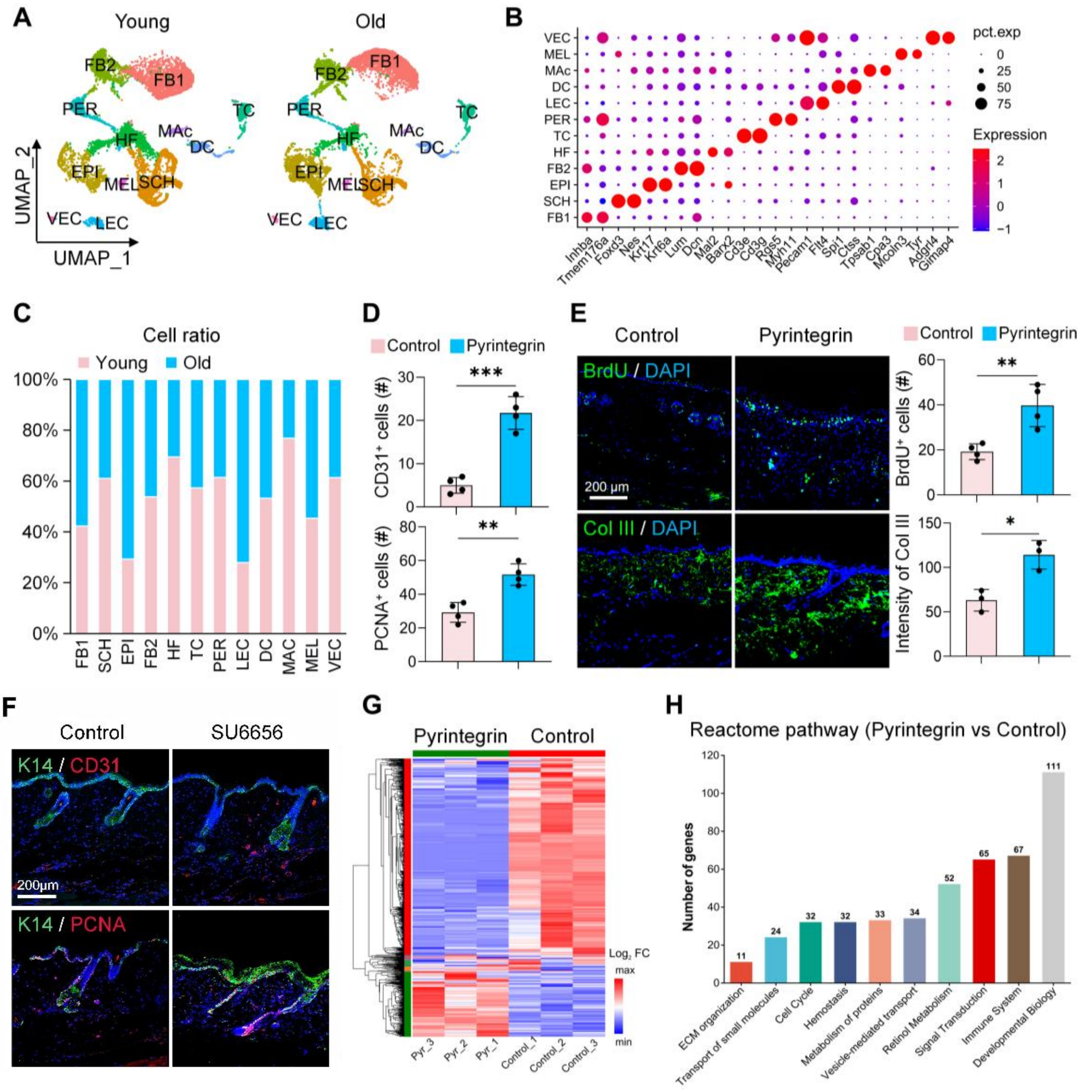


**Figure S3 Activates integrin receptors to promote aging skin rejuvenation.**

A. UMAP plot showing the distribution of single-cell subpopulations in the dorsal skin of young and aged mice.

B. Bubble plot showing marker genes of cell subpopulations in the dorsal skin of mice.

C. Stacked bar graph comparing the proportions of cells in scRNA-seq data from young and aged mice.

D. Quantitative analysis of CD31-positive cells (upper panel) and PCNA-positive cells (lower panel) in young Pyrintegrin-treated and control groups. n = 4, $^{**}p < 0.01$, $^{***}p < 0.001$.

E. Left: Representative immunofluorescence images showing the expression of BrdU and Col III in Pyrintegrin-treated and control groups. Right: Quantitative analysis

of BrdU-positive cell numbers and relative fluorescence intensity of Col III. N = 4, *p < 0.05, ***p < 0.001.

F. Representative immunofluorescence staining showing changes in K14, CD31, and PCNA expression in aged mouse skin of control versus SU6656 (ITGA9 activator)-treated groups, n = 3.

G. Heatmap showing the distribution of differentially expressed genes in RNA-seq analysis between pyrintegrin-treated and control groups.

H. Bar graph showing Reactome enrichment analysis terms of upregulated genes in the pyrintegrin group.

**Figure S4**

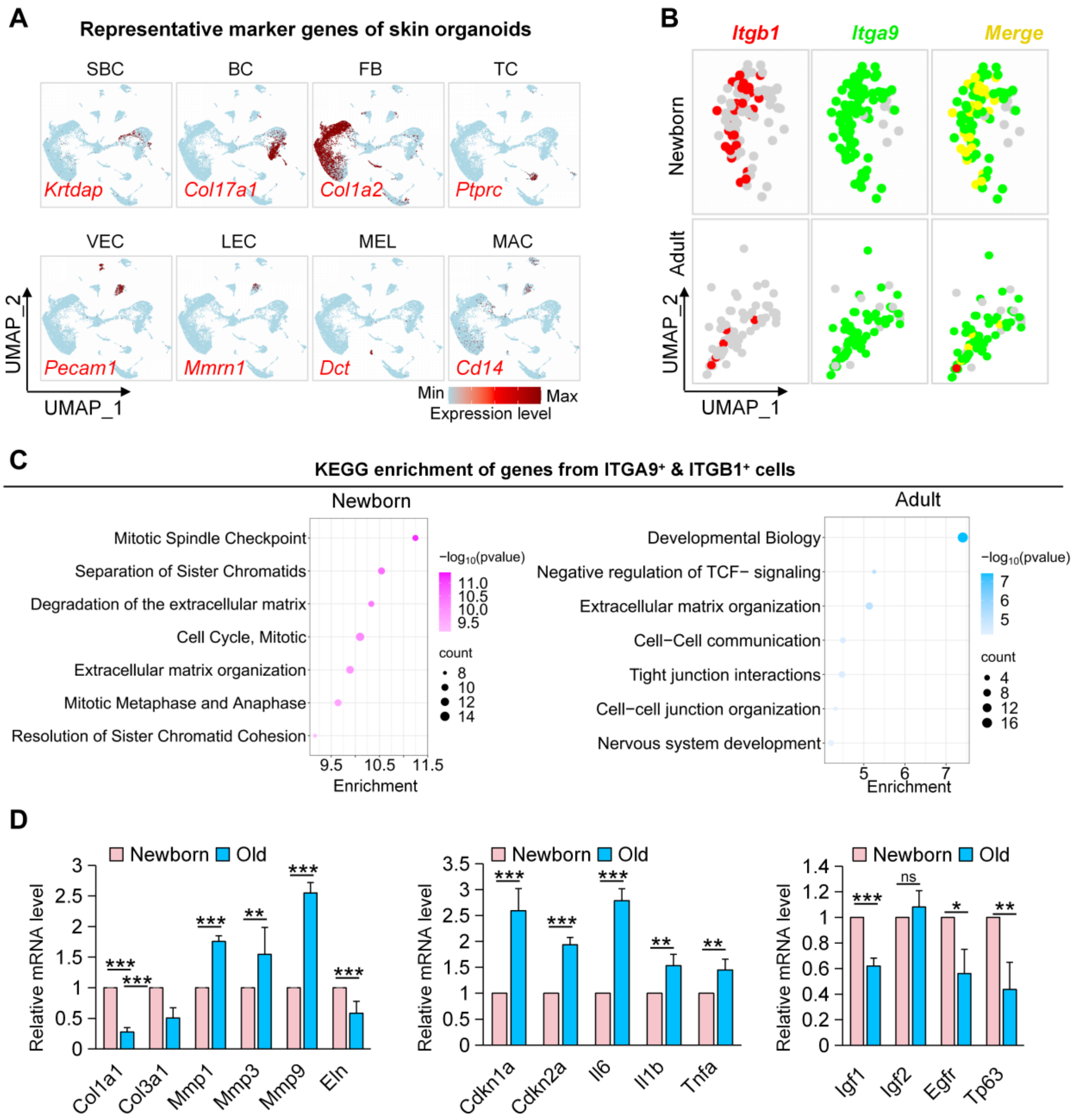


**Figure S4 Skin organoid system reveals that Itga9 and Itgb1 are associated with EC proliferation.**

A. Feature plot showing the expression of representative marker genes in scRNA-seq data of mouse skin organoids.

B. Feature plot comparing the co-expression of Itgb1 and Itga9 in endothelial cell subpopulations between newborn and adult skin organoids.

C. Enrichment analysis comparing the high-expression genes in cells co-expressing Itgb1 and Itga9 in endothelial cell subpopulations between newborn and adult skin organoids.

D. RT-qPCR analysis of differential expression in skin organoids comparing newborn

versus aged groups: ECM-related genes (left panel), SASP-associated genes (middle panel), and epidermal development-related genes (right panel). N = 3; *p < 0.05, **p < 0.01, ***p < 0.001; "ns" denotes non-significant differences.

**Figure S5**

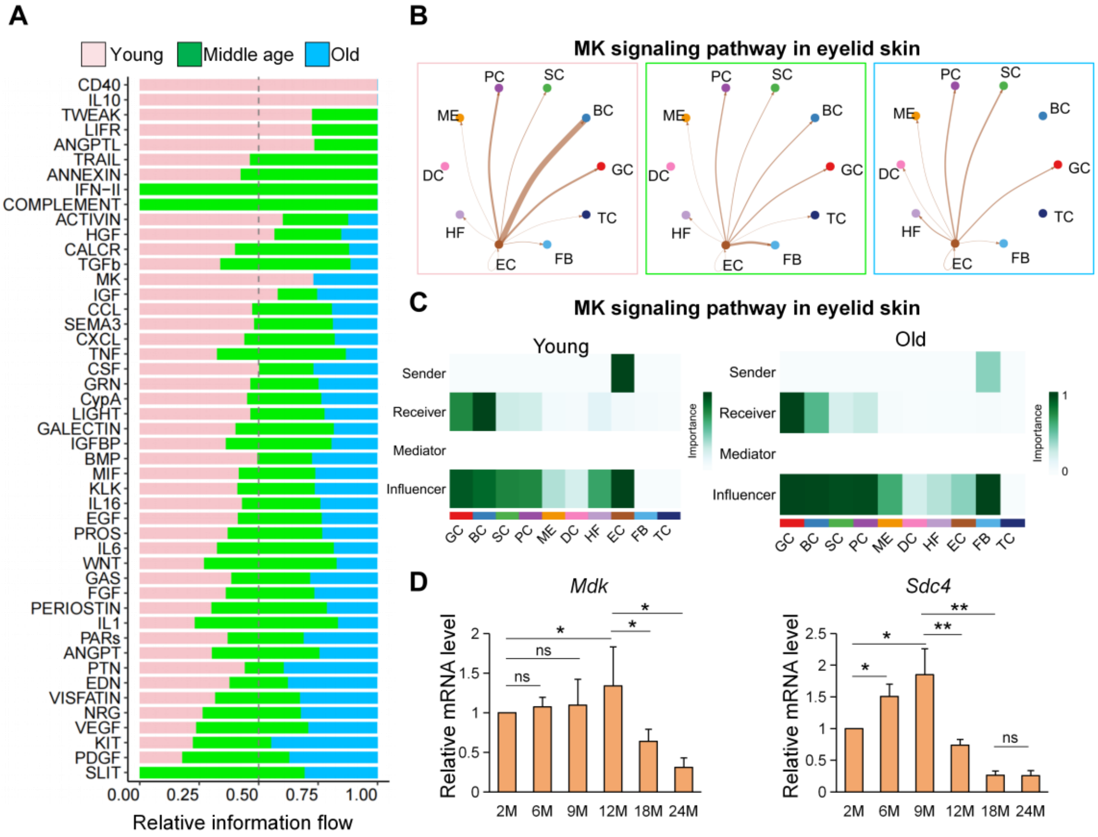


**Figure S5 CellChat analysis demonstrates enhancement of MDK-SDC4 signal between EC and BC.**

A. Comparison of interaction strength between VEC and BC based on the *Secreted* signaling pathway in young, middle-aged, and aged buttock skin.

B. Circle plot comparing the interaction strength of ECs as signal senders with other cell populations based on MK (midkine) signaling in young, middle-aged, and aged eyelid skin.

C. Importance analysis of senders, receivers, mediators, and influencers in the MK signaling pathway in young and aged eyelid skin.

D. RT-qPCR analysis of Mdk and Sdc4 expression levels in dorsal skin across age groups (2M, 6M, 9M, 12M, 18M, 24M). N = 3; *p < 0.05, **p < 0.01, ***p < 0.001; "ns" indicates non-significant differences.

## Figure S6

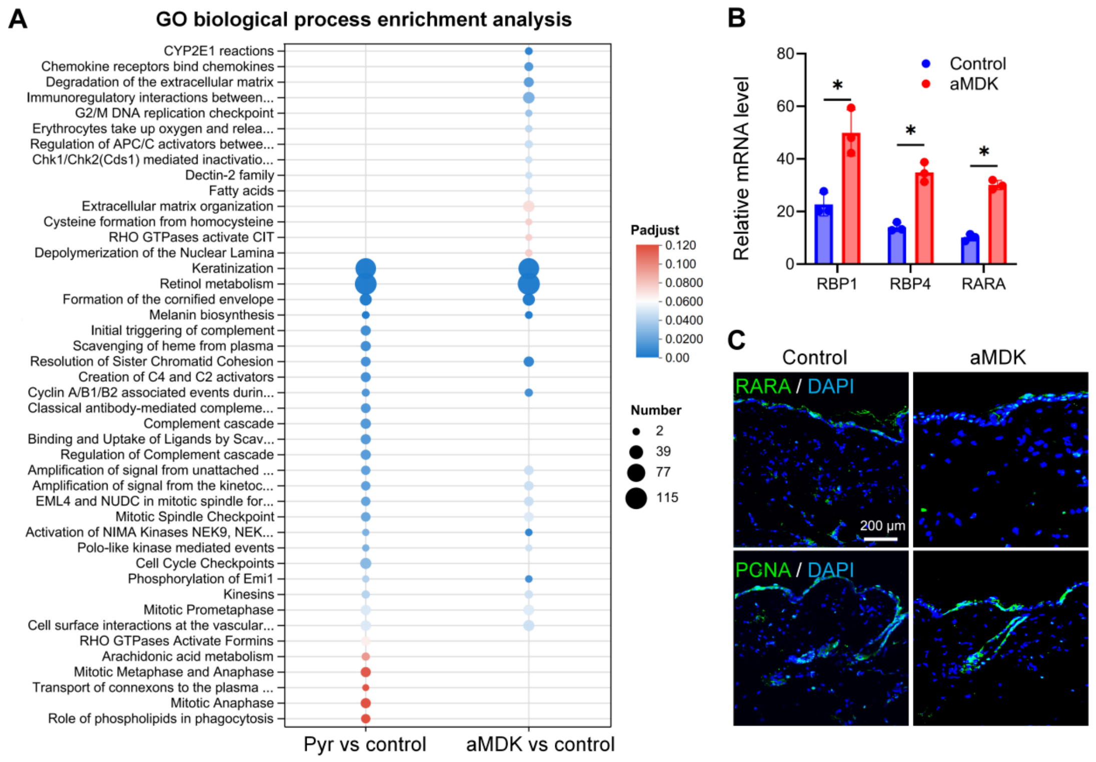


**Figure S6 MDK activates retinol metabolism signaling in epidermal cells.**

A. RT-qPCR analysis comparing the expression levels of Rbp1, Rbp4, and Rara in control and aMDK groups, n = 3, *$p < 0.05$.

B. Representative immunofluorescence images showing the expression levels of RARA and PCNA in control and aMDK groups.

C. Reactome enrichment analysis comparing the signaling pathways enriched by upregulated genes in Pyrintegrin and aMDK groups.

Figure S7

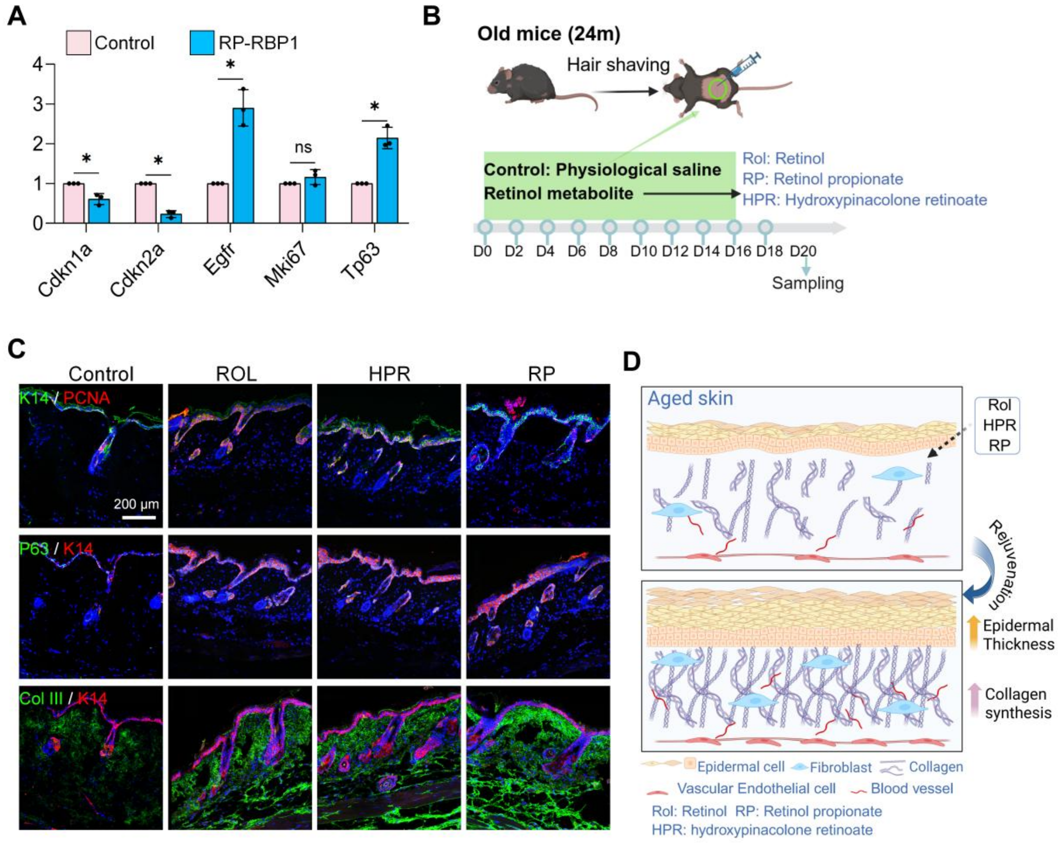


**Figure S7 RBP1 promotes the rejuvenation of aging skin organoids**

A. RT-qPCR analysis comparing the expression levels of senescence-related markers Cdkn1a and Cdkn2a, as well as epidermal growth-related genes Egfr, Mki67, and Tp63 in control and RP-RBP1 mouse skin, n = 3, *p < 0.05, "ns" means no significance.

B. Schematic of the experimental procedure for treating 24-month-old mice with retinol metabolite.

C. Representative immunostaining of epidermal regeneration markers in murine skin sections: basal keratinocyte markers (KRT14, TP63), proliferation marker PCNA, and extracellular matrix component Col III across experimental groups - untreated (Control), retinol-treated (ROL), hydroxypropyl retinamide (HPR), and retinol palmitate (RP). Biological replicates n = 3.

D. Schematic diagram illustrating retinoid-mediated rejuvenation effects in aged murine skin.

**Table S1** Primer Information

| Gene name | Forward | Reverse |
|---|---|---|
| Vegfa | CTGCCGTCCGATTGAGACC | CCCCTCCTTGTACCACTGTC |
| Vegfb | GCCAGACAGGGTTGCCATAC | GGAGTGGGATGGATGATGTCAG |
| Pdgfra | AGAGTTACACGTTTGAGCTGTC | GTCCCTCCACGGTACTCCT |
| Itga1 | CCTTCCCTCGGATGTGAGTCA | AAGTTCTCCCCGTATGGTAAGA |
| Robo1 | GAGCCTGCTCACTTTTACCTC | GGTCTGAAGGGTGTTCAACAAT |
| Rbp1 | CTGAGCAATGAGAATTTCGAGGA | GCGGTCGTCTATGCCTGTC |
| Rbp4 | AGTCAAGGAGAACTTCGACAAGG | CAGAAAACTCAGCGATGATGTTG |
| Rara | ATGTACGAGAGTGTGGAAGTCG | ACAGGCCCGGTTCTGGTTA |
| Col1a1 | GCTCCTCTTAGGGGCCACT | ATTGGGGACCCTTAGGCCAT |
| Col3a1 | CTGTAACATGGAAACTGGGGAAA | CCATAGCTGAACTGAAAACCACC |
| Mmp1 | AACTACATTTAGGGGAGAGGTGT | GCAGCGTCAAGTTTAACTGGAA |
| Mmp3 | GGCCTGGAACAGTCTTGGC | TGTCCATCGTTCATCATCGTCA |
| Mmp9 | TTAAAGACAGGCACTTTTGGCG | CCCTCGTATAGCCCAGAACT |
| Eln | TGTCCCACTGGGTTATCCCAT | CAGCTACTCCATAGGGCAATTTC |
| Cdkn1a | CCTGGTGATGTCCGACCTG | CCATGAGCGCATCGCAATC |
| Cdkn2a | CGCAGGTTCTTGGTCACTGT | TGTTCACGAAAGCCAGAGCG |
| Il6 | TAGTCCTTCCTACCCCAATTTCC | TTGGTCCTTAGCCACTCCTTC |
| Il1b | GAAATGCCACCTTTTGACAGTG | TGGATGCTCTCATCAGGACAG |
| Tnfa | CAGGCGGTGCCTATGTCTC | CGATCACCCCGAAGTTCAGTAG |
| Igf1 | CTGGACCAGAGACCCTTTGC | GGACGGGGACTTCTGAGTCTT |
| Igf2 | GTGCTGCATCGCTGCTTAC | CGGTCCGAACAGACAAACTG |
| Egfr | GCCATCTGGGCCAAAGATACC | GTCTTCGCATGAATAGGCCAAT |
| Tp63 | CCACAGTACACGAACCTGGG | TGGAAGGACACATCGAAGCTG |
| Mdk | GAAGAAGGCGCGGTACAATG | GAGGTGCAGGGCTTAGTCA |
| Sdc4 | TTTGCCGTTTTCCTGATCCTG | TTGCCCAAGTCGTAACTGCC |
| MDK(H) | CGCGGTCGCCAAAAAGAAAG | TACTTGCAGTCGGCTCCAAAC |
| SDC4(H) | GGACCTCCTAGAAGGCCGATA | AGGGCCGATCATGGAGTCTT |

**Table S2** Antibody information

| Antibody | Company | Cat # |
|---|---|---|
| BrdU | Abcam | Cat #ab6326 |
| CD31 | Servicebio | Cat #GB12063-100 |
| Collagen III | Proteintech | Cat # 22734-1-AP |
| E-cadherin | Beyotime | Cat # AF0138 |
| EGFR | Abcam | Cat # ab52894 |
| ITGB1 | Proteintech | Cat # 12594-1-AP |
| KRT14 | Boster | Cat # A01432 |
| Laminin | Abcam | Cat # ab11575 |
| MDK | Proteintech | Cat #11009-1-AP |
| NCL | Proteintech | Cat #83380-1-RR |

| | | |
|---|---|---|
| P63 | GeneTex | Cat # GTX102425 |
| PCNA | Abcam | Cat # ab29 |
| RARA | Proteintech | Cat #10331-1-AP |
| RBP1 | Abcam | Cat #ab307683 |
| RBP4 | Proteintech | Cat #11774-1-AP |
| RBP7 | Proteintech | Cat #14541-1-AP |
